# International Sport Forum of the Strength & Conditioning Society (SCS) and the European Sport Nutrition Society (ESNS)

**DOI:** 10.3390/sports8090128

**Published:** 2020-09-16

**Authors:** Pedro E. Alcaraz, Robert Csapo, Tomás T. Freitas, Elena Marín-Cascales, Anthony J. Blazevich, Antonio Paoli

**Affiliations:** 1 Research Center for High Performance Sport, Catholic University of Murcia, UCAM, 30107 Murcia, Spain; tfreitas@ucam.edu (T.T.F.); emarin@ucam.edu (E.M.-C.); 2 Faculty of Sports, Catholic University of Murcia, UCAM, 30107 Murcia, Spain; 3 Strength and Conditioning Society, 00118 Rome, Italy; robert.csapo@umit.at (R.C.); a.blazevich@ecu.edu.au (A.J.B.); antonio.paoli@unipd.it (A.P.); 4 Research Unit for Orthopaedic Sports Medicine and Injury Prevention, ISAG, UMIT, 6060 Hall, Austria; 5 NAR—Nucleus of High Performance in Sport, São Paulo 04753060, Brazil; 6 Centre for Exercise and Sports Science Research (CESSR), School of Medical and Health Sciences, Edith Cowan University, Joondalup 6027, Australia; 7 Department of Biomedical Sciences, University of Padova, 35122 Padova, Italy; 8 CIR-Myo, University of Padova, 35122 Padova, Italy; 9 European Sport Nutrition Society, 00118 Rome, Italy

**Keywords:** strength & conditioning, sport nutrition, high-performance, sport, exercise

## Abstract

On behalf of the Strength & Conditioning Society (SCS) and the European Sport Nutrition Society (ESNS), we are pleased to present the abstracts of the 2019 International Sport Forum on Strength & Conditioning & Nutrition, which took place in Madrid, Spain from November 15th–16th 2019. The meeting provided evidence-based education to advance the science and practice on the fields of sport nutrition, training, rehabilitation and performance. It also disseminated cutting-edge sport nutrition and strength and conditioning research, promoted the translation of basic science into the field and fostered the future of the field by providing young practitioners and researchers with the opportunity to present their findings through oral and poster communications, the abstracts of which can be found in this Special Issue of Sports. Renowned international and national speakers provided comprehensive updates, workshops and insights into novel scientific topics covering various areas of sport nutrition and strength and conditioning science. We were fortunate to have a wide range of speakers and presenters from all areas—strength training, conditioning to prevent injuries and improve performance, nutrition and supplementation for fitness and high-performance sports. A data-flash and poster session allowed for the presentation of the latest results of current research. Most importantly, the meeting provided ample opportunities to bring people together to discuss practical questions related to training and nutrition and plan scientific projects. With cutting-edge research and best practice in mind, this joint conference was an important means to pursue the missions of the SCS and ESNS. Rather than being a single event, the forum in Madrid was the starting point for a series of regular meetings on Strength & Conditioning & Nutrition to be held worldwide, so make sure to visit the websites of the SCS and ESNS and follow us on social media to receive updates and connect with our members. We proudly look back on an exciting, inspiring and informative meeting in Madrid!

## 1. Effects of Different Strategies for Reducing Neuromuscular Deficit Associated with Morning-Evening Performance Associated with Circadian Rhythms

López-SamanesAlvaro^1^*Moreno-PérezVictor^2^MorencosEsther^3^Salom-MorenoJaime^1^MuñozAlejandro^3^BerlangaLuis^1^ValadésDavid^1^Pérez-LópezAlberto^4^SedliakMilan^5^1School of Physiotherapy, Faculty of Health Sciences, Universidad Francisco Vitoria, 28223 Madrid, Spain; jaime.salom@ufv.es (J.S.-M.); luis.berlanga@ufv.es (L.B.); davidvaladesnavarro@icloud.com (D.V.)2Center for Translational Research in Physiotherapy, Department of Pathology and Surgery, Universidad Miguel Hernández, Elche, 03550 San Juan, Spain; vmoreno@goumh.umh.es3Exercise and Sport Sciences. Faculty of Health Sciences, Universidad Francisco de Vitoria, 28223 Madrid, Spain; esther.morencos@ufv.es (E.M.); alejandro.munoz@ufv.es (A.M.)4Department of Biomedical Sciences, Area of Sport and Physical Education, Faculty of Medicine and Health Sciences, University of Alcalá, 28805 Madrid, Spain; alberto_perez-lopez@hotmail.com5Faculty of Physical Education and Sports, Comenius University, 81469 Bratislava, Slovakia; milan.sedliak@uniba.sk*Correspondence: alvaro.lopez@ufv.es; Tel.: +34-91-709-14-00 (ext. 1955)Circadian rhythms (CR) are cyclic variations that take place in periods around 24 h. These CR affect sports performance, especially during morning exercise in unaccustomed athletes presenting 5–10% lower values in maximal strength and power values compared to evening values. The aim of this study was to compare different protocols for reducing the neuromuscular deficit associated morning-evening in neuromuscular performance. Methods: Seventeen sports science students (age: 22.1 ± 1.6 years), participated and performed six randomly strategies such as placebo AM (PLA AM), dynamic warm-up (DWU AM), music AM (MUS AM), caffeine AM (CAF AM), placebo PM (PLA PM) and caffeine PM (CAF PM). Afterwards each participant realized a neuromuscular test battery consisted of countermovement jump (CMJ), isometric handgrip strength (IHS), modified agility *t*-test (MATT) and sprint velocity 20 m (v20m). Results: DWU AM, CAFF AM, PLA PM and CAF PM produced higher CMJ performance against PLA AM (*p* = 0.001−0.048), CAF AM, PLA PM and CAF PM protocol produced lower values in MATT protocol against PLA AM (*p* = 0.015−0.047). Conclusions: CAF AM and DWU AM represented good strategies for reducing the neuromuscular differences in jump capacity against PLA AM values. In addition, CAFF AM for agility values respect PLA AM.chronobiologywarm-upergogenic aids

**Funding:** This research received no external funding.

## 2. Maximum Speed in Matches and Maximum Sprint Speed in Young Soccer Players: Effects of the Game Position

Cervantes-AguileraEsteban^1^*Ochoa-AhmedFernando A.^1^,^2^Avalos-AguilarRodolfo^1^González-CantúÁngel^2^Salas-FraireOscar^2^1Faculty of Sports Organization, Autonomous University of Nuevo Leon, San Nicolás de los Garza, Mexico; fernando.ochoahm@uanl.edu.mx (F.A.O.-A.); rodolfo.aval0s@hotmail.com (R.A.-A.)2Faculty of Medicine, Autonomous University of Nuevo Leon, San Nicolás de los Garza, Mexico; angel.gonzalezcnt@uanl.edu.mx (Á.G.-C.); ekosaar@gmail.com (O.S.-F.)*Correspondence: esteban.cervantesgl@uanl.edu.mx; Tel.: +52-442-23-23-782The aim of this study was to assess the relationship between peak match speed (PMS) and maximal sprinting speed (MSS) regarding playing position. MSS and absolute PMS (PMSAbs) were collected from 10 male youth soccer players (category U13). The best result from a 40 m sprint test was used to determine MSS. PMSAbs was recorded using a global positioning system (15 Hz GPSport, Canberra, Australia) and was also expressed as a percentage of MSS (PMSRel). The best result from the five matches recorded was taken to elicit PMSAbs. Sprint data were compared between playing positions. Results showed that all the players displayed a high value of PMSRel surpassing one hundred percent of the PMSAbs. On average, forwards recorded the best MSS (22.9 km/h ± 0.3), and center-midfielders showed the lowest (21.7 km/h ± 2.4). However, center-backs displayed on average the best PMSAbs and PMSRel (e.g., 28.1 km/h ± 0.4 and 124% ± 1%, respectively), while center-midfielders showed again the lowest results (e.g., 23.7 km/h ± 1.4 and 110% ± 5%, respectively). The findings showed that the maximum speed turned out to be dependent on the playing position, affecting the absolute and relative intensity of speed-related actions during matches.sprintyouthsoccer

**Funding:** This study received no external funding.

## 3. Gut Microbiota as a Potential Factor of Weight Gain Limitations in Athletes

BielikViktor^1^*BalážVladimír^1^HricIvan^1^PenesováAdela^2^AugustovičováDušana^1^UgrayováSimona^1^ŠoltysKatarína^3^1Department of Biological and Medical Science, Faculty of Physical Education and Sport, Comenius University, Bratislava, Slovakia2Institute of Clinical and Translational Research Biomedical Center, Slovak Academy of Science, Bratislava, Slovakia; adela.penesova@savba.sk3Department of Microbiology and Virology, Faculty of Natural Science, Comenius University, Slovakia; katarina.soltys@uniba.sk*Correspondence: viktor.bielik@uniba.sk; Tel.: +421-220-669-931As interest in the human microbiome grows, there is an increasing number of studies that can be used to test numerous hypotheses across human populations. Obesity has been shown to be associated with an alteration of gut bacterial community types in human. In contrast, little is known about gut microbiome of lean athletes with positive caloric balance. The aim of this study was to characterize gut microbiota composition of athletes with positive energy balance, yet unable to gain weight. In this study, the stools of lean athletes and control group were collected. Massive parallel sequencing of amplicons 16S rDNA gene was used to assess the differences and the taxonomic composition of the gut microbiota. Food monitoring was used to distinguish athletes with poor dietary habits and inadequate caloric intake and evaluate energy balance. A physical activity questionnaire and basal metabolic rate were used to calculate total daily energy expenditure. The sequencing results showed relatively abundant bacteria with significantly different distributions between normal and case samples. Although detailed functional roles or mechanisms of these bacteria are needed for further validation, the results provide new insights into bacterial communities in the gut of athletes with weight-gain limitations.microbiome16S rDNAobesityphysical activity

**Funding:** This research was funded by the Slovak Research and Development Agency grant no. APVV-17-0099.

## 4. Session-Rating-Perceived Exertion Following Resistance Training with Different Interset Strategies

VieiraDenis^1^,^2^*SantosCatharina^1^AvelinoMatheus^1^NascimentoDahan^1^,^3^OliveiraSamuel^1^BottaroMartim^2^1Department of Physical Education, UDF—University Center, Brasília, Brazil2College of Physical Education, University of Brasilia—UnB; martim@unb.br3Graduation Program of Physical Education, Catholic University of Brasilia—UCB, Brasília, Brazil*Correspondence: denisclvieira@hotmail.com; Tel.: +55-61-993999866The aim of this study was to compare the effects of interset static stretching, interset blood flow restriction and passive rest intervals in internal training load. Nine resistance-trained men performed three knee-extension protocols with interset static stretching (ISS), interset blood flow restriction (IBFR) or passive rest interval (PAS). Both protocols consisted of five sets of concentric knee extension until achieving the fixed total work for each set (1st set: 2.200 J; 2nd set: 2.200 J; 3rd set: 2.000 J; 4th set: 2.000 J; 5th set: 1.600 J). During the 90-s rest intervals, the individuals performed 40 s of static stretching for knee extensor muscles, blood flow restriction by a cuff in proximal region of the leg or remain seated at rest. Twenty minutes after each RT session, the individuals reported SRPE through CR-10 RPE scale. A repeated measure ANOVA reported that the SRPE were similar between all RT sessions with different interset rest intervals (*p* = 0.06; ISS = 5.89 ± 1.90; IBFR = 5.33 ± 2.45; PAS = 4.56 ± 1.81). Thus, our results suggest that interset static stretching, interset blood flow restriction and passive rest interval produce the same internal training load during RT.internal loadblood flow restrictioninterset static stretching

**Funding:** This research was funded by the University of Brasilia and the UDFF—University Center.

## 5. Mechanical Differences between Heavy- vs. Light-Load Resistance Training in Older People

Rodriguez-LopezCarlos^1^,^2^Navarro-CruzRoberto^1^,^2^AlcazarJulian^1^,^2^Baltasar-FernandezIvan^1^,^2^CsapoRobert^3^AraIgnacio^1^,^2^AlegreLuis M.^1^,^2^*1GENUD Toledo Research Group, Universidad de Castilla-La Mancha, Toledo, Spain2CIBER of Frailty and Healthy Aging (CIBERFES), Madrid, Spain3Research Unit for Orthopaedic Sports Medicine and Injury Prevention, ISAG, University for Health Sciences, Medical Informatics and Technology, Hall, Austria*Correspondence: luis.alegre@uclm.comThis study evaluated the mechanical differences of a heavy- vs. light-load power-oriented resistance training in older people. Using a crossover design, fifteen well-functioning older volunteers (six women; 73.6 ± 3.8 years) completed two volume×load-matched ballistic resistance-training sessions with heavy (HL: 6 × 6 × 80% 1-RM) and light loads (LL: 6 × 12 × 40% 1-RM) on a horizontal leg press exercise. Mechanical variables (work, force, velocity and power), as well as intra-set neuromuscular fatigue (i.e., relative losses in force, velocity and power) were analyzed. More concentric mechanical work was performed in the LL training session, compared with HL (36.2 ± 11.2%; *p* < 0.001). Greater concentric force (35.2 ± 7.6%; *p* < 0.001) during HL, higher concentric velocity (41.0 ± 12.7%, *p* < 0.001) and a trend towards higher concentric power (7.2 ± 18.9%, *p* = 0.020) were found for LL. Relative velocity losses were similar in both sessions (≈10%); however, relative force losses were only found in LL (7.4 ± 6.5%, *p* = 0.005). In older people, volume × load-matched ballistic resistance training using light loads may, therefore, represent a stronger stimulus driving training adaptations that are contingent upon mechanical work and power production compared to heavy-load training. Relative losses in force and power should be monitored in addition to velocity losses during ballistic resistance training.intensityagingpowervelocitywork

**Funding:** This research was funded by the Ministerio de Economía y Competitividad of Spain, Grant Number DEP2015-69386-R and BES-2016-077199.

## 6. Power Loss as Indicator for Prescribing and Controlling High-Intensity Interval Training in Cycling

Salazar-MartínezEduardo^1^,^2^*LópezJavier Sola^3^1Universidad Pablo de Olavide, Sevilla, Spain2Centro de Estudios Universitarios Cardenal Espínola CEU, Sevilla, Spain3Universidad Loyola, Sevilla, Spain*Correspondence: esalmar@upo.esBackground: The aim of this study was to evaluate the influence of two different high-intensity interval training (HIIT) training interventions (10% power loss vs. 20% power loss) in cycling performance.; (2) Methods: Six trained cyclists (VO_2max_ = 58.57 ± 2.21) were randomized in two HIIT groups (10% loss (n = 3) vs. 20% loss (n = 3)). Each group completed eight weeks of training (15 HIIT sessions). Cycling performance was evaluated before and after training intervention using an incremental exercise test (VO_2max_, PPO, VT1, VT2) and 20 min time trial (P20, W/kg P20). HIIT training was prescribed using the power output at VO_2max_ (PPO). Each subject performed “n” intervals at PPO until the power loss were equal to 10% or 20%; (3) Results: Both groups improved their cycling performance (PPO and W/kg P20) after training intervention (*p* < 0.05). No differences were found between groups in any variable studied. No differences were found between the training stress score (TSS) (10% loss = 2352 ± 1511.03 and 20% loss = 5145.66 ± 2702.6) and intensity factor (IF) (10% loss = 0.77 ± 0.6 and 20% loss = 0.76 ± 0.6) between groups (4) Conclusions: The present data could suggest that the loss of power during HIIT intervals could be a good indicator for prescribing and controlling HIIT training in trained cyclists.HIITpowercycling

**Funding:** This research received no external funding.

## 7. Acute Physiological Responses to Different High-Intensity Interval Training Regimes and an Intensive Continuous Training in Active Students

PanascìMarco^1^,^2^*FaelliEmanuela^2^,^3^FerrandoVittoria^1^,^2^BisioAmbra^2^,^3^RuggeriPiero^2^,^3^1Department of Neuroscience, Rehabilitation, Ophthalmology, Genetics, Maternal and child Health, University of Genoa, Genoa, Italy2Centro Polifunzionale di Scienze Motorie, University of Genoa, Genoa, Italy3Department of Experimental Medicine, Section of Human Physiology, University of Genoa, Genoa, Italy*Correspondence: marco.panasci87@gmail.comThis study investigated the physiological and psychological responses of a high-intensity continuous training (CT) and three high intensity interval training (HIIT) protocols. Fifteen active students (age: 21.0 ± 1.07 years, height: 173.9 ± 8.97 cm, body mass: 64.81 ± 12.93 kg, VO_2max_ 48.1 ± 7.49 mL/kg/min) were enrolled in this study. VO_2max_ and MAS were assessed through an incremental treadmill test. Each participant performed on separate days and in a randomized order a high intensity CT, at 95% MAS, until exhaustion and 10–20, 30–30, 50–30, lasting for 16 min, alternating phase at 95% MAS and recovery at 40% MAS. VO2, VCO2, RER, HR, T@VO_2max_, [La] + and RPE were measured in each session. VO_2peak_ was significantly higher in CT versus 10–20 (p < 0.001) and 30–30 trainings (*p* < 0.05). Furthermore, VO_2peak_ in 10–20 was significantly lower than in 30–30 and 50–30 (*p* < 0.001). T@VO_2max_ in 50–30 was significantly higher than in CT (*p* < 0.01) and 10–20 trainings (*p* < 0.001). Additionally, 10–20 T@VO_2max_ was significantly lower than in 30–30 (p < 0.05). RER values in CT was significantly higher than those in 10–20 (*p* < 0.001) and 30–30 (*p* < 0.01). A total of 10–20 RER values were significantly lower than those in 50–30 (always *p* < 0.01). In CT HRmax was significantly higher than those in 10–20 and 30–30 (*p* < 0.001 and *p* < 0.01, respectively), whereas 10–20 HRmax was lower than 30–30 (*p* < 0.05) and 50–30 (*p* < 0.01). [La]+ was significantly higher in CT than in 10–20 (*p* < 0.001) and 30–30 (*p* < 0.01). Finally, RPE associated with CT was significantly higher than those in 30–30 (*p* < 0.01) and 10–20 (*p* < 0.001). Our results showed that 50–30 HIIT gave an optimal stimulus to elicit both maximal cardiovascular and peripheral adaptations, as, under this training condition, subjects spent the longest time in their “red zone”, which means at least 90% of their maximal oxygen uptake, compared to other training modalities.HIITphysiological responsesRPE

**Funding:** This research received no external funding.

## 8. Body Composition of Ice Hockey Players throughout the Season

KokindaMarek^1^*KičuraDaniel^2^KandráčRóbert^1^LenkováRút^1^1Faculty of Sports, University of Presov, Presov, Slovakia2HC Kosice, Kosice, Slovakia; daniel.kicura@icloud.com*Correspondence: marek.kokinda@unipo.skThe purpose of this study was to analyze the changes in body composition as a result of ice hockey training and matches during the preseason and in-season. Participants were 24 senior ice hockey players from an elite ice hockey team: 2 goaltenders, 8 defensemen and 14 forwards: mean age = 27.8 years. The research methodology was divided into three stages: (1) summer off-ice training, (2) on-ice training and (3) in-season. We analyzed body composition using the direct segmental multi-frequency bioelectrical impedance analysis in each of the stages. Body composition analyses during both preseason and in-season were conducted once per month always before and after a league game. Parameters measured and assessed included (1) body weight (BW), (2) total body water (TBW), (3) body fat mass (BFM), (4) skeletal muscle mass (SMM), (5) basal metabolic rate (BMR) and (6) visceral fat area (VFA). The results showed that the summer off-ice training had a significant effect on body composition (*p* < 0.05). The SMM increased and BFM decreased, on average, by 1.3 kg and 1.5 kg, respectively. These changes caused the increase in BMR by 41 kcal and decrease in VFA by 16.6 cm^2^. The most significant changes were found for defensemen. The differences in pregame and postgame body composition showed that players experienced changes in body composition, especially in BW and BFM (*p* < 0.05). From the scientific point of view, an interesting finding is that the postgame visceral fat area increased. Visceral fat area significantly correlated with the active ice time (*p* < 0.05).trainingInBodyperformance capacity

**Funding:** This research was funded by the Scientific Grant Agency of the Ministry of Education, Science, Research and Sport of the Slovak Republic and Slovak Academy of Sciences, Grant Number VEGA 1/0573/18 entitled, “The function of trunk muscle strength in the development of athletic performance and injury prevention”.

## 9. Effect of Hip Joint Angle on Acute Concentric Knee Extension Work output and Muscle Swelling

BottaroMartim^1^*DouradoMarco^1^UgliaraLucas^1^VieiraDenis^1^,^2^1College of Physical Education, University of Brasilia, Brasília, Brazil2Department of Physical Education, UDF—University Center, Brasilia, Brazil; denisclvieira@hotmail.com*Correspondence: martim@unb.br; Tel.: +55-61-31072522The purpose of this study was to investigate the effects of hip joint position during knee extension exercise on work output and muscle swelling of the rectus femoris (RF) and vastus lateralis (VL). Twelve resistance-trained men performed two concentric knee extension protocols in fully extended (0°) and 90° flexed hip positions. Both protocols consisted of five sets of 10 repetitions at 60°∙s^−1^. Muscle thickness (MT) of the middle and proximal RF and VL were assessed by an ultrasound. The amount of total work was significantly greater in the flexed (9540.89 ± 1213.67 J) when compared to the extended hip position (8791.90 ± 821.36 J). MT of middle RF increased (*p* < 0.05) for both flexed (28.36 ± 3.99 to 32.91 ± 3.74 mm) and extended hip position (26.95 ± 3.44 to 29.94 ± 4.16 mm). There was a significant (*p* < 0.05) increase in MT on both hip positions of the proximal VL. However, MT increased significantly (*p* < 0.05) in the middle VL (29.20 ± 4.57 to 31.22 ± 5.05 mm) only in the flexed position. Therefore, these results indicate that flexed hip position (i.e., seated) yield greater knee extensors training volume and muscle swelling in the VL.knee extensiontotal workmuscle swelling

**Funding:** This research was funded by the University of Brasilia and by the Brasília Research Foundation (FAPDF).

## 10. Modulation Inflammatory State after Eight Weeks Chronic Cardiose^®^ Intake in Trained Cyclists

Martínez-NogueraFrancisco Javier^1^*Marín-PagánCristian^1^Carlos-VivasJorge^2^Campoy-De HaroLaura^1^Marín-CascalesElena^1^,^3^Pérez-HernándezAndrés^1^AlcarazPedro E.^1^,^3^1UCAM Research Center for High Performance Sport, Catholic University of Murcia, UCAM, 30107 Murcia, Spain2Health, Economy, Motricity and Education Research Group (HEME), Faculty of Sport Sciences, University of Extremadura, Cáceres, Spain3Faculty of Sport Sciences, Catholic University of Murcia, UCAM, 30107 Murcia, Spain*Correspondence: fjmartinez3@ucam.eduHesperidin (Cardiose^®^ (NLT 85% 2S-hesperidin) supplement) is a flavonoid that may decrease the inflammatory state. The objective of this study was to assess the effects of eight-week supplementation with 500 mg of Cardiose^®^ (n = 20) compared to placebo (n = 20) in inflammatory markers in male cyclists. A randomized and double-blinded study was performed. Participants came to the lab twice for pre-post tests: (1) maximal test on a cycloergometer to determine metabolic zones and power output (Fat_max_, ventilatory threshold one and two and maximal power output (MPO)) and (2) steady step test, with three venous blood extractions: at baseline, after MPO and at recovery. In the steady step test, significant differences were shown at baseline in MCP-1; in IL-6 and MCP-1 after recovery; and in MCP-1 AUC for Cardiose^®^. In the same test, significant differences were observed at baseline in TNFα and MCP-1; in TNFα and MCP-1; and in TNFα and MCP-1 AUCs after MPO for placebo. The intake of 500 mg/day of Cardiose^®^ improved the inflammatory rest state and acute post-exercise recovery. These changes with hesperidin supplementation optimize recovery strategies for athletes following strenuous exercise.inflammationexercise2S-hesperidin

**Funding:** This research received no external funding.

## 11. Chronic Cardiose^®^ Intake Improves Functional Threshold Power and Base–Acid Balance in Trained Cyclists

Martínez-NogueraFrancisco Javier^1^*Marín-PagánCristian^1^Carlos-VivasJorge^2^Campoy-De HaroLaura^1^Marín-CascalesElena^1^,^3^Pérez-HernándezAndrés^1^AlcarazPedro E.^1^,^3^1UCAM Research Center for High Performance Sport, Catholic University of Murcia, UCAM, 30107 Murcia, Spain2Health, Economy, Motricity and Education Research Group (HEME), Faculty of Sport Sciences, University of Extremadura, Cáceres, Spain3Faculty of Sport Sciences, Catholic University of Murcia, UCAM, 30107 Murcia, Spain*Correspondence: fjmartinez3@ucam.eduThe objective of this study was to assess the chronic effects of eight-week supplementation with 500 mg of Cardiose^®^ in performance and metabolic markers. A randomized, double-blind study was performed. Forty male cyclists were assigned to two groups: Cardiose^®^ and placebo. Participants came to the lab twice for pre-post measurements: (1) maximal test on cycle ergometer to determine metabolic zones, power output (at Fat_max_, Note we adjusted your verb tense here. Please ensure your intended meaning has been retained. 1, VT2 and VO_2max_) and estimated functional threshold power (FTP); and (2) steady step test on cycle ergometer and finger blood sample were collected at the end of each step and analyzed with a gasometer. Significant pre-post differences were found in FTP for Cardiose^®^ during the incremental test. Significant pre-post increases were found in SBC, HCO_3_^−^, ABE and SBE at VT1, 2nd Fat_max_ and during recovery. A significant decrease was also observed in La^+^ at 1st Fat_max_, 2nd Fat_max_ and during recovery for Cardiose^®^. However, significant pre-post increases in HCO3− and SBE at VT2 and significant decrease in La+ and 1st Fat_max_ in placebo was found. Chronic intake of Cardiose^®^ improved base–acid balance and increases power output at FTP in the cyclists. These changes improved athlete performance in high-intensity efforts.lactate2S-hesperidinperformance

**Funding:** This research received no external funding.

## 12. Influence of Strength and Power Capacities on Change-of-Direction Speed and Deficit in Elite Team-Sport Athletes

FreitasTomás T.^1^,^2^,^3^,^4^,^5^*AlcarazPedro E.^1^,^3^PereiraLucas A.^2^,^4^ArrudaAdemir F. S.^5^GuerrieroAristide^5^AzevedoPaulo H. S. M^4^LoturcoIrineu^2^,^4^,^6^1UCAM Research Center for High Performance Sport, Catholic University of Murcia, UCAM, 30107 Murcia, Spain2NAR—Nucleus of High Performance in Sport, São Paulo, Brazil3Faculty of Sport Sciences, Catholic University of Murcia, UCAM, 30107 Murcia, Spain4Department of Human Movement Sciences, Federal University of São Paulo, São Paulo, Brazil5Brazilian Rugby Confederation, São Paulo, Brazil6University of South Wales, Pontypridd, Wales, UK*Correspondence: tfreitas@ucam.eduRecently, change of direction (COD) deficit has been proposed as a complementary way to evaluate COD ability as a separate quality, by isolating the acceleration capability of the athlete. To investigate the influence of maximum strength and power levels on COD deficit in team-sport athletes, seventy-eight elite players (soccer, n = 46; rugby, n = 32) performed: 20-m linear sprint and Zigzag COD tests, half-squat 1-repetition-maximum (HS 1RM) and jump-squat peak power (JS PP). Using median split analysis, athletes were divided into higher and lower HS 1 RM and higher and lower PP JS groups. Magnitude-based inferences were used to analyze the between-group differences. Athletes in the high strength and power groups outperformed their weaker and less powerful counterparts in the 20-m sprint and zigzag COD tests. Moreover, stronger and more powerful athletes displayed greater COD deficits. The data indicate that players with superior strength-power capabilities tend to be less efficient at changing direction relative to maximum sprinting speed—despite being faster in linear trajectories. It appears that current strength and power training practices in team sports are potentially not ideal for increasing an athlete’s aptitude to efficiently utilize his neuromuscular capabilities during COD maneuvers.performancedirectional changesagility

**Funding:** This research received no external funding.

## 13. Differences Among Physical Performance Indices of Professional and Semi-Professional Greek Soccer Players

SpyrouKonstantinos^1^*FreitasTomás T.^1^,^2^,^3^,^4^AlcarazPedro E.^1^,^4^1UCAM Research Center for High Performance Sport, Catholic University of Murcia, UCAM, 30107 Murcia, Spain2NAR—Nucleus of High Performance in Sport, São Paulo, Brazil3Department of Human Movement Sciences, Federal University of São Paulo, São Paulo, Brazil4Faculty of Sport Sciences, Catholic University of Murcia, UCAM, 30107 Murcia, Spain*Correspondence: kspyrou@ucam.eduThe aim of this study was to determine the parameters that distinguish top male Greek soccer players from their lower level counterparts. Thirty-five male soccer players participated in the study, in a sample composed by professional first division players (elite; n = 16) and semi-professional fourth division players (sub-elite; n = 19). Fat mass, sit and reach test, isokinetic strength of the knee extensor and flexor muscles at 60°·s^−1^ angular velocity, squat jump (SJ), countermovement jump (CMJ) and countermovement jump with free hands height (CMJf) were assessed. Additionally, VO2_max_ and maximum heart rate (HR_max_) on a treadmill test were measured. Professional players obtained significantly better results than the semi-professional athletes in SJ did (ES = 1.11, *p* = 0.00), CMJ (ES = 1.21, *p* = 0.00) and CMJf (ES = 0.97, *p* = 0.01). Moreover, no statistically significant, but moderate meaningful differences were found for fat mass (ES = 1.03) and small for V02_max_ (ES = 0.46) and HR_max_ (ES = 0.43), when comparing elite and sub-elite athletes. No significant differences were found between playing level for H/Q ratio in absolute and relative torque at 60°·s^−1^ (ES = 0.04) and sit and reach test (ES = 0.11). Based on these findings, neuromuscular performance outcomes such as SJ, CMJ and CMJf, may be the most important determinant factors selective Greek male professional and semi-professional soccer players.fitnesseliteteam sports

**Funding:** This research received no external funding.

## 14. Complex Training: Comparison of Time under Tension Focus vs. Traditional Training during the Conditioning Activity on Performance Measures Using Sub-Maximal Loads

SimpsonChris*PatersonStephenTallentJamieSt Mary’s University, London, UK; stephen.paterson@stmarys.ac.uk (S.P.); Jamie.tallent@stmarys.ac.uk (J.T.)*Correspondence: chris@fit-4-purpose.comThe purpose of this study was to identify long-term effects of complex training with a time under tension (TUT) vs. traditional lifting (TRAD) method during the conditioning activity (CA) on performance measures over a five-week intervention. Second, whether using submaximal loads will increase performance (3 RM back squat, counter-movement jump (CMJ) and 10-m sprint (S10)), while comparing genders. A total of 25 university soccer players (14 women (w) and 11 men (m)) were divided into TUT group (n = 13) or TRAD group (n = 11). A control group (n = 8) conducted in pre- and posttests. Groups completed two sessions per week, matched for all training variables and identical complex set (back squat and CMJ), with an optimal load used on the CA. The CA differed between groups (TRAD—2 s per repetition and TUT—5 s per repetition). Statistical significance in strength levels for both experimental groups (*p* < 0.05) with performance increases at 14.1% vs. 14.3%. Male TUT strength increase 16%. Increases shown in CMJ (1.6 vs. 1.7%). Male TUT displayed greatest improvement (2.8%). Female TUT group experienced a positive improvement in S10 time (−1.1%). In conclusion, both methods elicited the potentiation effects associated with complex training and enhanced both strength and CMJ performance.complexTUTstrength

**Funding:** This research received no external funding.

## 15. Warm-up Performance and Physiological Effects During a 20-min Cycling Time Trial

AlejoLidia B.^1^*Gil-CabreraJaime^1^ValenzuelaPedro L.^2^,^3^Montalvo-PérezAlmudena^1^TalaveraEduardo^1^Moral-GonzálezSusana^1^Clemente-SuárezV. J.^1^Barranco-GilDavid^1^LuciaAlejandro^1^1Faculty of Sport Sciences, Universidad Europea de Madrid, Madrid, Spain2Department of Systems Biology, University of Alcalá, Madrid, Spai3Department of Sport and Health, Spanish Agency for Health Protection in Sport (AEPSAD), Madrid, Spain*Correspondence: lidia.brea@universidadeuropea.es; Tel.: +349-12115381The most commonly performed warm-up is arguably a short bout of sub-maximal exercise, e.g., ~60–70% maximum oxygen uptake (VO_2max_). However, it has been proposed that the addition of brief, task-specific bursts of high-intensity exercise to this standard warm-up would provide further ergogenic benefits. This effect, known as post-activation potentiation (PAP), is thought to facilitate a greater force production capacity. The aim of this study was to analyze the performance and physiological effects of different warm-up protocols on endurance performance. Fifteen male cyclists (35 ± 9 years; VO_2max_: 66.4 ± 6.8 mL·kg·min-1) participated on this randomized study. They performed a 20-min cycling time-trial preceded by either no warm-up, a standard-warmup (10 min at 60% VO_2max_) or a warm-up designed to elicit PAP (five minutes at 60% VO_2max_ followed by three 10-s all-out sprints interspersed with a 90-s rest). Performance (jump ability, power output (PO)), perceptual (rating of perceived exertion (RPE)) and physiological (VO2, muscle oxygenation (SmO2), heart rate variability (HRV), blood lactate and skin temperature) responses were measured. An enhanced jump ability (*p* < 0.001) was observed after standard-warmup (9.7 ± 4.7%) and PAP-warmup (12.9 ± 6.5%), but not with no-warmup (−1.9 ± 4.8%, *p* = 0.074). Both standard-warmup (−7.9 ± 14.2%, *p* = 0.027) and PAP-warmup (−20.3 ± 24.7%, *p* = 0.006)—but not no-warmup (−1.7 ± 10.5%, *p* = 0.366)—resulted in a decreased HRV. Participants started the trial (minutes 0–3) at a higher PO and VO2 with PAP-warmup compared to the other conditions (*p* < 0.05), but no differences were overall found for the mean PO, RPE, VO2 or SmO2 during the trial (all *p* > 0.05). Warming-up enhanced neuromuscular performance (i.e., jumping) and induced a shift towards sympathetic modulation. The inclusion of brief sprints resulted in a higher initial intensity during the subsequent time trial.performancepreconditioningexercise

**Funding:** This research received no external funding.

## 16. Effects of Menstrual Cycle Phase on Strength Performance in Team-Sport Athletes Using Oral Contraceptives

ReifAstrid^1^*HaiderPatricia^1^VidottoClaudia^2^TschanHarald^1^WessnerBarbara^1^TriskaChristoph^1^,^3^1Centre for Sport Science and University Sports, University of Vienna, Vienna, Austria2Labor Vidotto; labor@labor-vidotto.at3Austrian Institute of Sport Medicine, Vienna, Austria; info@sportmedizin.or.at*Correspondence: astrid.reif@univie.ac.at; Tel.: +43-1-4277-48873The oral contraceptive pill (OCP) is frequently used by female athletes. Some, but not all studies report direct effects on strength performance between contraceptive pill phases. Therefore, the objectives of this research were to determine whether strength performance varies depending on hormone balance throughout the oral contraceptive cycle. Eight female handball players (age: 24.6 ± 3.6 yrs; body mass: 66.0 ± 4.0 kg; body stature: 1.68 ± 0.05 m) were recruited for this study. Participants were tested on the second or third day and on day 16–17 of menstrual cycle (follicle and luteal phase). Participants had to perform two isokinetic (60°/s) knee flexion and extension repetitions each and two isometric (five seconds at 60°) extension repetitions with each leg using Isomed 2000 (Ferstl GmbH, Regensburg, Germany) interspersed by three min passive rest. Before each test, venous blood samples were taken to test the hormonal balance of estradiol and progesterone. No significant differences were found for isokinetic peak torque extension (p = 0.701; d = 0.05 and *p* = 0.837; d = 0.04 for left and right, respectively) and flexion (*p* = 0.673; d = 0.05 and *p* = 0.955; d = 0.02 for left and right, respectively). For isometric peak torque (*p* = 0.861; d = 0.03 and *p* = 0.451; d = 0.27 for left and right, respectively) and time to peak torque (*p* = 0.862; d = 0.10 and *p* = 0.739; d = 0.16 for left and right, respectively) no significant differences were found. Significant differences were found for peak rate of torque development on the left leg (*p* = 0.046; d = 0.55), but not on the right leg (*p* = 0.544; d = 0.15). The present data suggest that there are no physiological relevant differences in strength parameters between phases of oral contraceptive cycle.handballcontraceptionfemale athletes

**Funding:** This research received no external funding.

## 17. Lower-Limb Asymmetry in Older People: Differences in Muscle Function, Sex and Their Relation to Physical Function

Sanchez-MartinCoral^1^Rodriguez-LopezCarlos^1^AlcazarJulián^1^CsapoRobert^2^AraIgnacio^1^,^3^AlegreLuis M.^1^,^3^*1GENUD Toledo Research Group, Universidad de Castilla-La Mancha, Toledo, Spain2Research Unit for Orthopaedic Sports Medicine and Injury Prevention, University for Health Sciences, Medical Informatics and Technology, Hall, Austria3CIBER of Frailty and Healthy Aging (CIBERFES), Madrid, Spain*Correspondence: luis.alegre@uclm.es; Tel.: +34-925-268-800 (ext. 5506)Lower-limb asymmetries in muscle function may increase the risk of falls among older women. However, no data are available in men. The purpose of this study was to clear whether this effect is sex-specific and to assess the relationship between muscle asymmetries and other measures of physical function. Participants (thirty-three well-functioning older volunteers) completed a force–velocity relationship test and a 1-RM test in the unilateral leg press exercise (Technogym, Italy) with a linear position transducer mounted (Chronojump, Spain) to assess maximum dynamic strength, maximum muscle power and movement velocity. Physical function was assessed by means of the SPPB. Lower-limb asymmetry (%) was calculated for both 1-RM and Pmax. No differences were found between 1-RM and Pmax asymmetry values. Furthermore, no sex-related differences were identified for 1-RM or Pmax asymmetry values. Finally, no associations were found between physical function and asymmetry outcomes. Both maximal muscle strength and power muscular asymmetries were similar in elderly men and women. Asymmetries in muscle function were not related to other measures of physical function in healthy older adults.resistance trainingagingimpairment

**Funding:** This research was funded by MINECO/FEDER, EU, grant number DEP2015-69386-R and by CIBERFES and FEDER funds from the European Union, grant number CB16/10/00477.

## 18. Influence of Footwear on Spatiotemporal Parameters and Foot Kinematics while Running Using RunScribe™ Footpods

Jaén-CarrilloDiego^1^*García-PinillosFelipe^2^Cartón-LlorenteAntonio^1^Roche-SeruendoLuis E.^1^1Universidad San Jorge, Villanueva de Gállego, Zaragoza, Spain; acarton@usj.es (A.C.-L.); leroche@usj.es (L.E.R.-S.)2Department of Physical Education, Sports and Recreation, Universidad de La Frontera, Temuco, Chile; fegarpi@gmail.com*Correspondence: djaen@usj.es; Tel.: +34-678-63-89-67Unlike today—when most runners use modern shoes with cushioning and support—people in the past would run either barefoot or simple shoes. The aim of this study was to analyze the influence of footwear on running spatiotemporal parameters, foot-strike pattern and foot pronation velocity in amateur runners using the RunScribe™ foot pods. A total of 51 healthy subjects, 34 men and 17 women, (age: 27.84 ± 7.01 years; height: 172.88 ± 7.92 cm; body mass: 67.89 ± 10.9 kg; 10 km time: 47.85 ± 6.87 min) run at a self-selected comfortable velocity under the shod and unshod condition on a treadmill. Data were recorded using the RunScribe™ sensor (triaxial accelerometer and gyroscope) over three min for both conditions and mean values analyzed. Maximum pronation velocity, contact time, flight time, step frequency, stride length and foot-strike pattern (1) were compared between measures by using ANOVA and Cramer’s V analyses. Significant differences were found between shod and unshod conditions for all the variables analyzed—CT (*p* < 0.001), FT (*p* < 0.001), SF (*p* < 0.001), SL (*p* < 0.001) MPV (*p* < 0.001) and FSP (*v* < 0.2). Running spatiotemporal parameters and running kinematics, in terms of MPV and FSP, differ between footwear conditions within the same subjects.barefootkineticspronation velocitysensorswearables

**Funding:** This research received no external funding.

**Acknowledgments:** The authors would like to thank all participants.

## 19. Influence of Oral Contraceptives and the Menstrual Cycle on Endurance Performance in Competitive Handball Players

TriskaChristoph^1^,^2^*HaiderPatricia^1^VidottoClaudia^3^WessnerBarbara^1^ReifAstrid^1^1Centre for Sport Science and University Sports, University of Vienna, Vienna, Austria2Austrian Institute of Sport Medicine, Vienna, Austria3Labor Vidotto, Vienna, Austria*Correspondence: christoph.triska@univie.ac.at; Tel.: +43-1-4277-48867Recently, assessing the effects of different phases of the menstrual cycle on strength and endurance performance has gained interest in scientific works. Changes in the female hormones during the menstrual cycle are suggested to influence endurance as well as strength and speed abilities. When using a monophasic combination pill as an oral contraceptive two different phases can be described: (1) high estradiol and gestagen concentration during 21 days of taking the pill and (2) low estradiol and gestagen concentration during 7 days of absence of the pill. Therefore, the aim of this study was to assess differences in endurance performance, i.e., $\overset{\cdot }{V}$O_2_ and heart rate (HR) at 2.78 m∙s^−1^; absolute und relative $\overset{\cdot }{V}$O_2max_; HR_max_; maximal blood lactate concentration (La); maximal aerobic speed (MAS) between phase 1—represented by high concentrations in estradiol and gestagen—and phase 2—represented by low concentrations in estradiol and gestagen. Eight competitive female handball players (age: 24.6 ± 3.6 yrs; body mass: 66.0 ± 4.0 kg; body stature: 1.68 ± 0.05 m) volunteered to participate in this study. Participants were reported to the laboratory on two occasions. One test was conducted on Day 2 or Day 3 of the menstrual cycle (phase 2) and the other on day 16 or 17 of the menstrual cycle (phase 1). The order of the test was randomized. Before each test 7 mL venous blood samples were taken in order to analyze for hormonal concentration and the body mass of the participants was measured. A graded exercise test on a motorized treadmill (Saturn, h/p/cosmos, Traunstein, Germany) starting at 1.67 m∙s^−1^ with an increment of 0.28 m∙s^−1^ every 1 min was used to assess endurance performance. Throughout the test respiratory gases were measured breath-by-breath using a mobile gas analyzer (MetaMax3B-R3, Cortex Biophysik GmbH, Leipzig, Germany). Immediately and three min after terminating the treadmill test 20 µL blood samples were taken from the earlobe in order to assess [La_max_] and subsequently analyzed (Biosen S_Line, Barleben, Germany). HR was measured using a heart rate sensor (H7, Polar Electro Oy, Kempele, Finland) connected to the gas analyzer. Differences between phases were assessed using a paired-samples *t*-test and effect sizes were evaluated using the Cohen’s *d*. Significance was accepted as *p* < 0.05. No significant differences were found for $\overset{\cdot }{V}$O_2_ and HR at 2.78 m∙s^−1^, absolute and relative $\overset{\cdot }{V}$O_2max_, HR_max_, [La_max_], MAS and body mass (*p* = 0.178 − 1.000), and the effect sizes were of a small order (*d* = 0.03 − 0.28). Significant differences, however, were found for gestagen and estradiol concentrations between the phases (*p* < 0.05). The findings of this study demonstrate statistically insignificant differences in endurance exercise performance within the menstrual cycle in handball players taking a monophasic combination pill. Therefore, the monthly bleeding has no detrimental nor beneficial effects in handball players.
team-sportscontraceptionfemale athletes

**Funding:** This research received no external funding.

## 20. Effects of Caffeine Ingestion on Physical Performance in Elite Women Handball Players: A Randomized, Controlled Study

MuñozAlejandro^1^,^2^*López-SamanesÁlvaro^3^Pérez-LópezAlberto^4^Aguilar-NavarroMillán^1^Moreno-HerederoBerta^1^Rivilla-GarcíaJesús^3^González-FrutosPablo^1^Pino-OrtegaJosé^5^MorencosEsther^1^Del CosoJuan^6^1Exercise and Sport Sciences. Faculty of Health Sciences, Universidad Francisco de Vitoria, 28223 Madrid, Spain; millan.aguilar@ufv.es (M.A.-N.); b.moreno@ufv.es (B.M.-H.); p.gfrutos.prof@ufv.es (P.G.-F.); esther.morencos@ufv.es (E.M.)2Faculty of Physical Activity and Sports Sciences (INEF). Universidad Politécnica de Madrid (UPM), Madrid, Spain3School of Physiotherapy, Faculty of Health Sciences, Universidad Francisco Vitoria, 28223 Madrid, Spain; alvaro.lopez@ufv.es4Department of Biomedical Sciences, Area of Sport and Physical Education, Faculty of Medicine and Health Sciences, University of Alcalá, 28805 Madrid, Spain; alberto_perez-lopez@hotmail.com5Department of Physical Activity and Sport Science, Sport Science Faculty, University of Murcia, San Javier, Spain; pepepinoortega@gmail.com6Centre for Sport Studies. Rey Juan Carlos University, 28943 Fuenlabrada, Madrid, 25 Spain; juan.delcoso@urjc.es*Correspondence: alejandro.munoz@ufv.es; Tel.: +34-91-709-14-00The aim of this study was to investigate the effects of acute caffeine intake on aspects of physical performance of elite female handball players. Fifteen elite female handball players participated in a randomized and doubled-blind study. In two different trials, participants either ingested a placebo or three milligrams of caffeine (mg/kg/bm). Participants underwent a battery of neuromuscular tests and performed a simulated handball game. Compared with the placebo, CAFF increased ball velocity in all BT (*p* = 0.021 − 0.044; ES = 0.39 − 0.49), strength in IHS (350.8 ± 41.2 vs. 361.6 ± 46.1 N, *p* = 0.034; ES = 0.35) and CMJ height (28.5 ± 5.5 vs. 29.8 ± 5.5 cm; *p* = 0.006; ES = 0.22). Moreover, CAFF decreased running time in the SV (4.9 ± 0.2 vs. 4.8 ± 0.3 s; *p* = 0.042; ES = −0.34). In the simulated game, CAFF increased the frequency of accelerations (18.1 ± 1.2 vs. 18.8 ± 1.0 number/min; *p* = 0.044; ES = 0.54), decelerations (18.0 ± 1.2 vs. 18.7 ± 1.0 number/min; *p* = 0.032; ES = 0.56) and body impacts (20 ± 8 vs. 22 ± 10 impacts/min; *p* = 0.032; ES = 0.30). Ingestion of caffeine improved aspects of handball-specific physical performance in female elite handball players.handballcaffeineperformance

**Funding:** This study was partially funded by Universidad Francisco de Vitoria and Banco Santander by grants.

## 21. Nutritional Supplement Practices of Beach Volley Players of the Italian National Championship: A Cross-Sectional Study

AmatoriStefano*SistiDavideLantignottiMichelaGervasiMarcoBertuccioliAlexanderZeppaSabrina DonatiCalavalleAnna RitaRocchiMarco B. L.Department of Biomolecular Sciences, Service of Biostatistics, University of Urbino Carlo Bo, Piazza Rinascimento 7, 61029 Urbino, Italy; davide.sisti@uniurb.it (D.S.); m.lantignotti@campus.uniurb.it (M.L.); marco.gervasi@uniurb.it (M.G.); alexander.bertuccioli@uniurb.it (A.B.); sabrina.zeppa@uniurb.it (S.D.Z.); anna.calavalle@uniurb.it (A.R.C.); marco.rocchi@uniurb.it (M.B.L.R.)*Correspondence: s.amatori1@campus.uniurb.it; Tel.: +39-349-2115150The ingestion of some nutritional supplements can enhance sport performance, but often, scientific studies for specific disciplines are lacking. The conditions in which beach volley matches are played make it a unique discipline, but no studies have been conducted to investigate the efficacy of specific supplements in this context. The purpose of this cross-sectional study was to evaluate the supplementation practices of high-level beach volley athletes participating in the Italian National Championship. A total of 88 athletes participated in the study. Sociodemographic characteristics, nutritional supplement use, motivations for use and sources of information were collected using an anonymous pretested online questionnaire. Latent class analysis (LCA) was used to highlight a profile of athlete from supplement items. Chi-squared test was used to compare different variables. Most frequently used supplements were vitamins, calcium, iron, protein, amino acids, carbohydrate, caffeine, glutamine and creatine. LCA showed three different classes of athletes characterized by different behaviors in supplement consumption: no supplements (LC_1_), all supplements included in the study (LC_2_), only some classes of supplement (LC_3_). Nutritionists were the main sources of information for the supplements, followed by the Internet and teammates. Most athletes use supplements to improve performance and recovery and to prevent nutritional deficiencies. Our data showed that a large number of athletes do not take any supplementation, but another class reported to take almost every supplement listed in the study. Greater reliance by athletes on nutritionists providing evidence-based support could influence the supplements selection, thus improving the effects on performance and health.athletesdietary supplementsteam sports

**Funding:** This research received no external funding.

## 22. Acute Effects of Different Loading Conditions on Kinematic and Leg Stiffness in Rugby Players

Carlos-VivasJorge^1^*ZabaloySantiago^2^FreitasTomás T.^1^,^3^Pareja-BlancoFernando^2^LoturcoIrineu^3^ComynsTom^4^Gálvez-GonzálezJavier^2^AlcarazPedro E.^1^1UCAM Research Center for High Performance Sport, Catholic University of Murcia, UCAM, 30107 Murcia, Spain2Physical Performance & Athletic Research Center, Universidad Pablo de Olavide, Sevilla, Spain3NAR—Nucleus of High Performance in Sport, São Paulo, Brazil4Department of Physical Education and Sport Sciences, University of Limerick, Limerick, Ireland*Correspondence: jorge.carlosvivas@gmail.comAim: The aim of this study was to compare the acute effects of unresisted and resisted sprint training (RST) on kinematic and leg stiffness (Kleg) during a 30-m sprint in rugby players. Methods: Twelve amateur rugby players performed. (1) 30-m sprint with 0%, 20%, 40%, 60% and 80% BM to obtain individual equations for calculating velocity loss (Vloss) conditions; (2) Sprint trials with 0%, 10%, 30% and 50% Vloss with ~4 min of rest. Kinematics was assessed using two slow-motion cameras from a sagittal plane and Kinovea software. Kleg, stride length (SL), stride frequency (SF), contact time (CT), flight time (FT) and trunk angle were calculated at touchdown. Results: Between-load comparisons revealed increases on trunk angle (small to very large) and CT (trivial to very large) and decreases in FT, SL and Kleg (trivial to very large) comparing 10%, 30% and 50% vs. 0% Vloss for acceleration phase. Similarly, increases on trunk angle (large to very large) and CT (small to nearly perfect) and decreases on FT (trivial to nearly perfect), SL (small to nearly perfect), SF (trivial to moderate) and Kleg (trivial to nearly perfect) comparing 10%, 30% and 50% vs. 0% Vloss for maximum-velocity phase. Conclusion: RST using loads heavier than 10% Vloss caused significant changes in kinematic and Kleg in rugby players.movement patternspeed trainingteam sports

**Funding:** This research received no external funding.

## 23. Acute Effects of Different Loading Conditions on Muscle Activation in Amateur Rugby Players

Carlos-VivasJorge^1^*ZabaloySantiago^2^FreitasTomás T.^1^,^3^Pareja-BlancoFernando^2^LoturcoIrineu^3^ComynsTom^4^Gálvez-GonzálezJavier^2^AlcarazPedro E.^1^1UCAM Research Center for High Performance Sport, Catholic University of Murcia, UCAM, 30107 Murcia, Spain2Physical Performance & Athletic Research Center, Universidad Pablo de Olavide, Sevilla, Spain3NAR—Nucleus of High Performance in Sport, São Paulo, Brazil4Department of Physical Education and Sport Sciences, University of Limerick, Limerick, Ireland*Correspondence: jorge.carlosvivas@gmail.comAim: The aim of this study was to compare the acute effects of unresisted and resisted sprint training (RST) on muscle activation during 30-m sprint in rugby players. Methods: Twelve amateur rugby players performed. (1) 30-m sprint with 0%, 20%, 40%, 60% and 80% BM to obtain individual equations for calculating velocity loss (Vloss) conditions; (2) Sprint trials with 0%, 10%, 30% and 50% Vloss with ~4 min of rest. To assess muscle activity, EMG signal was obtained from biceps femoris (BFlh), gastrocnemius (GAS), gluteus (GM) and rectus femoris (RF) using Noraxon Software. Electrodes were placed following SENIAM guidelines. Peak EMG activity was considered. Results: Acceleration phase comparisons showed decreases in GAS comparing 50% vs. 0% Vloss (trivial to very large) and BFlh (trivial to large) comparing 30% and 50% vs. 0% Vloss. In contrast, increases on GLU (trivial to very large) comparing 10% and 30% vs. 0% Vloss and RF (trivial to moderate) comparing 30% and 50% vs. 0% Vloss were found. Maximum-velocity phase comparisons revealed increases in GAS comparing 50% vs. 0% Vloss (trivial to small) and RF (small to very large) comparing 30% and 50% vs. 0% Vloss. In contrast, decreases in BF (trivial to very large) were observed comparing 30% and 50% vs. 0% Vloss. Conclusion: RST using loads heavier than 30% Vloss caused important changes in muscle activation patterns in rugby players.electromyographyspeed trainingteam sports

**Funding:** This research received no external funding.

## 24. Effects of Fatigue on Running Kinematics of Trained Long-Distance Athletes

Cartón-LlorenteAntonio^1^*Roche-SeruendoLuis E.^1^Jaén-CarrilloDiego^1^Marcen-CincaNoel^1^García-PinillosFelipe^2^1Universidad San Jorge, Zaragoza, Spain; leroche@usj.es (L.E.R.-S.); djaen@usj.es (D.J.-C.); nmarcen@usj.es (N.M.-C.)2Department of Physical Education, Sports and Recreation. Universidad de La Frontera, Temuco, Chile; fegarpi@gmail.co*Correspondence: acarton@usj.es; Tel.: +34-615635708The aim of this study was to assess the influence of fatigue on running spatiotemporal parameters, footstrike angle and impact and maximal pronation velocity in trained runners using the RunScribe™ foot pod. A total of 22 trained men (age: 34 ± 7.52 years; training: 41.73 ± 15.64 km/week; 10 km time: 37.22 ± 54.78 seg) run at his predicted maximal velocity for a 60 min effort. Three min before and after the fatiguing test, data were recorded at a constant velocity of 12 km/h using a treadmill. Significant differences were found between rested and fatigued conditions for: CT (*p* < 0.001), FT (P < 0.007), SA (*p* < 0.001), impact (*p* < 0.002), shock Gs (*p* < 0.003). No differences were found for: SF (*p* < 0.246), SL (*p* < 0.263) MPV (*p* < 0.478) and Braking Gs (*p* < 0.828). Our findings suggest that fatigue induces changes in running spatiotemporal parameters as well as running kinematics, in terms of contact and fly time, shock, impact and stride angle within the same test velocity. Stride frequency, stride length and maximal pronation velocity are not expected to vary if the velocity is maintained. endurancebiomechanicsinjuries

**Funding:** This research received no external funding.

## 25. Six-Week Multicomponent Exercise Program Using Cluster Set Configuration Improves Physical Function, Frailty and Muscle Power in Older Adults

SotoH.^1^,^2^*Baltasar-FernandezI.^1^,^2^Martín-BraojosS.^3^UcetaM. I.^3^AlegreL.^1^,^2^AraI.^1^,^2^García-GarcíaF. J.^2^,^3^Alfaro-AchaA.^2^,^3^Losa-ReynaJ.^1^,^2^,^3^1GENUD Toledo Research Group, Universidad de Castilla-La Mancha, Toledo, España2CIBER of Frailty and Healthy Ageing (CIBERFES), Madrid, España3Hospital Virgen del Valle, Complejo Hospitalario de Toledo, Toledo, España*Correspondence: hector.soto@gmail.comThe aim of this study was to describe the effects of a six-week supervised concurrent exercise program on physical function and frailty. This was a quasi-experimental, non-randomized controlled intervention in (pre)frail (2.7 ± 0.8 frailty phenotype criteria) older adults (82.4 ± 4.00 years). Physical function (short physical performance battery (SPPB)), frailty (Fried criteria) and muscle power (5-STS muscle-power test) were assessed at baseline and after six weeks. The intervention group (INT; n = 6) performed concurrent training twice weekly (power training: 3–4 sets of 8–12 reps/ 10 s rest each 2 reps/one min rest inter-sets at 30–50% F0; and HIIT: combining habitual and maximal gait speed). INT improved their habitual gait speed (0.53 ± 0.16 vs. 0.71 ± 0.14 m∙s−1; *p* < 0.05), balance (25.1 ± 4.5 vs. 27.4 ± 4.2 s; *p* < 0.05), SPPB (5.8 ± 1.6 vs. 8.67 ± 1.6; *p* < 0.05), Fried criteria (2.7 ± 0.8 vs. 1.3 ± 1.6; *p* < 0.05) and muscle power (159.8 ± 66.7 vs. 194.9 ± 66.8 W; *p* < 0.05). Additionally, two subjects out of three that did not stand-up from a chair were able to stand up after the exercise program while no changes in CON. Results showed that six weeks of a power-based concurrent exercise program using a cluster set configuration could be a good strategy to improve physical function in older people.agingmuscle powerfrailtycluster

**Funding:** This work was funded by the Biomedical Research Networking Center on Frailty and Healthy Aging (CIBERFES) and FEDER funds from the European Union (CB16/10/00477 and CB16/10/00456T)

## 26. Influence of Exergaming on Motor Competence, Lower Limb Lean Mass and Strength of Overweight and Obese Children

Comeras-ChuecaCristina^1^,^2^,^3^Gonzalez-AgüeroAlejandro^1^,^2^,^3^Villalba-HerediaLorena^1^,^2^,^3^Lozano-BergesGabriel^1^,^2^,^3^Marín-PuyaltoJorge^1^,^2^,^3^Matute-LlorenteAngel^1^,^2^,^3^Vicente-RodriguezGerman^1^,^2^,^3^CasajúsJosé Antonio^1^,^2^,^3^*1GENUD (Growth, Exercise, NUtrition and Development) research group, University of Zaragoza, Zaragoza, Spain2Faculty of Health and Sport Science (FCSD, Ronda Misericordia 5, 22001-Huesca, Spain), Department of Physiatry and Nursing, University of Zaragoza, Zaragoza, Spain3Red Española de Investigación en Ejercicio Físico y Salud en Poblaciones Especiales (EXERNET), Zaragoza, Spain*Correspondence: joseant@unizar.es; Tel.: +34-622896119Childhood obesity is an important public health problem characterized by poor motor competence and muscularity when involving weights. The aim of this study was to examine the influence of exergaming on motor competence (MC), lower limb lean mass and strength and investigate the relationships between strength variables and motor competence. Children (10.1 ± 0.8 years old) who were overweight or obese participated in a five-month exergaming intervention combined with exercise. MC was evaluated using the test for gross motor development–3 (TGMD-3), lower limbs lean mass was measured by dual-energy X-ray absorptiometry and lower limbs strength was assessed by performing counter movement jump test (CMJ) and a maximal isometric knee extension test. Dependent *t*-tests were used to examine the training effects and the relationship between MC and strength variables were studied. Results showed that MC, lower limbs lean mass, jump height and maximal isometric strength improved after intervention (p < 0.01) and there was a positive correlation between CMJ and TGMD-3 score. Exergaming combined with exercise showed a positive effect on lower limbs strength and lean mass and MC in overweight and obese children. CMJ seems to have a motor component that should be considered.exergameschildrenobesitymotor competencelower limbs strengthlower limbs lean mass

**Funding:** This research was funded by Ministerio de Economía, Industria y Competitividad (DEP2017-85194-P). Cristina Comeras Chueca received a Grant from de Government of Aragón (Spain).

## 27. Comparison of the Load–Velocity Relationship between Men and Women in Bench Press Exercise

Cornejo-DazaPedro J.^1^*WalkerSimon^2^HäkkinenKeijo^2^Pareja-BlancoFernando^1^1Physical Performance & Sports Research Center, Faculty of Sport Sciences, Universidad Pablo de Olavide, Sevilla, España2Faculty of Sport and Health Sciences, University of Jyväskylä, Jyväskylä, Finland*Correspondence: pjcordaz@gmail.comThe objective was to examine the differences between load–velocity relationships in men and women in bench press (BP) exercise, comparing three velocity variables: mean velocity (MV), mean propulsive velocity (MPV) and peak velocity (PV). Twenty-five men and 25 women performed a progressive loading test up to 1 RM for individual determination of the full load–velocity relationship in BP measured with a linear velocity transducer. The values of MV, MPV and PV for each %1RM, were obtained from the individual’s linear adjustments from approximately 30% 1 RM onwards, in increments of 5%. A strong relationship was found between MPV (R^2^ = 0.912, SEE = 0.085 m·s^−1^; R^2^ = 0.911, SEE = 0.066 m·s^−1^), MV (R^2^ = 0.911, SEE = 0.079 m·s^−1^; R^2^ = 0.910, SEE = 0.063 m·s^−1^) and PV (R^2^ = 0.874, SEE = 0.156 m·s^−1^; R^2^ = 0.868, SEE = 0.114 m·s^−1^) and% 1 RM in the load–velocity relationship in men and women. Men attained statistically higher velocity values (in three velocity variables) than women in light- to moderate-loads (<80% 1RM) (*p* range: <0.001‒0.05). The differences in the load–velocity relationships highlight the relevance of employing different equations for each sex or using the individual load–velocity relationships.load–velocity relationshipbench press exercisestrength training

**Funding:** Please add: This research received no external funding

## 28. Muscle Toning: Terminological Reflection

AriasCristian*MontenegroCristian Yanez DanielPinzonCarolFundación Universitaria del Area Andina, Bogotá, Colombia; cyanez@areandina.edu.co (C.Y.); dmontenegro8@areandina.edu.co (D.M.); cviolet@areandina.edu.co (C.P.)*Correspondence: carias39@estudiantes.areandina.edu.co; Tel.: +313-255-50-29In the field of physical exercise and fitness, the term of “muscular toning” is used to refer to obtaining “stiffness in the skeletal fibers” following exercise and/or load in strength training (resistance, hypertrophy and maximum strength). This term has no scientific basis in the literature, but is still used by coaches, doctors, athletes, instructors and gym users as synonymous with neuromuscular adaptations. (1) Background: This aim of this study was to define the term “muscle toning” due to the lack of scientific evidence in the field of exercise and sports sciences. (2) Methods: This literature review includes 30 scientific articles indexed in databases such as Pubmed, NCBI and academic google, using search terms or keywords such as: muscle toning, muscle strength, muscle strength, hypertrophy and strength resistance; (3) Results: No scientific articles were found in the databases consulted that defined the term “muscle toning”. Within the selection process, physiological adaptations are reaffirmed as in an increase in nerve impulses from the central nervous system to the peripheral one, thus achieving an increase in the sarcoplasmic content and myofibrillar size, which are generated by the training of the patient’s strength, but that differ from the term used in question; (4) Conclusions: There is no objective definition of the concept “muscle toning”. Adaptations of neuromuscular order are established, but do not define the use of the term within the field of physical exercise and fitness, but that claim to argue fibrillar growth, strength gain and sometimes an increase in size are defined as hypertrophy.muscular toningskeletal fibersresistancehypertrophymaximum strength

**Funding:** This research received no external funding.

**Acknowledgments:** We thank to teacher Robert John Mc Bride for assistance in constructing and comments on the manuscript.

## 29. Effects of High-Velocity Versus Traditional High-Effort-Based Resistance Training On Muscle Strength, Hypertrophy and Functional Performance in Elderly Women

BarbalhoMatheus^1^SouzaDaniel^1^*CoswigVictor Silveira^2^GentilPaulo^1^1Faculdade de Educação Física e Dança, Universidade Federal de Goiás, Goiânia, Brazil2Faculdade de Educação Física, Universidade Federal do Pará, Castanhal, Brazil*Correspondence: daniel_souza86@hotmail.comBackground: The aim of this study was to compare the effects of high velocity resistance training (HVRT) and traditional resistance training (TRT) on functional performance, muscle strength and hypertrophy in elderly women. Methods: One hundred and three elderly women were randomly divided into a PRT group (n = 50, 65.75 ± 3.26 years, 164.1 ± 3.48 cm, body mass 70.19 ± 3.77 kg) and a TRT group (n = 53, 68.38 ± 5.01 age, 164.3 ± 3.10 cm, body mass 68.54 ± 4.55 kg). Both groups performed a similar full-body RT twice per week. HVRT performed muscle action with maximum intended movement velocity and predefined repetitions, while TRT used controlled muscle action until momentary muscle. Results: After a 24-week training program, both groups obtained similar improvements in functional tests (*p* < 0.05). TRT group obtained higher increases in muscle hypertrophy measures (*p* < 0.05), whereas HVRT obtained higher increases in isometric strength (*p* < 0.001). Conclusion: Both resistance-training methods provided similar improvements on functional performance and dynamic muscle strength in older women, whereas the findings regarding muscle hypertrophy and isometric strength were dissimilar between groups. Therefore, while HVRT is a viable alternative when high efforts are not recommended, TRT may be prescribed to promote optimal gains in muscle size.resistance trainingagingfunctionality

**Funding:** This research received no external funding.

## 30. Effect of High-Intensity Interval Training and Intermittent Fasting on Body Composition and Leg Dynamic Performance in Active Women

García-De FrutosJosé Manuel^1^GunnarssonThomas P.^2^FiorenzaMatteo^2^Rubio-AriasJacobo Á.^3^Orquín-CastrillónFrancisco J.^1^Martínez-RodríguezAlejandro^4^*1Universidad Católica San Antonio de Murcia, Catholic University of Murcia, UCAM, 30107 Murcia, Spain; defrutos60@gmail.com (J.M.G.-D.F.), forquin@ucam.edu (F.J.O.-C.)2University of Copenhagen, Denmark; tgunnarsson@nexs.ku.dk (T.P.G.); maf@nexs.ku.dk (M.F.)3Polytechnic University of Madrid, Spain; ja.rubio@upm.es4University of Alicante, Alicante, Spain*Correspondence: amartinezrodriguez@ua.esIntermittent fasting is considered an alternative to daily calorie restriction. The objective is to create a net reduction in energy intake that makes it lower than energy expenditure, thus creating a state of negative energy balance and inducing weight loss. A single-group crossover design was used to compare the effects of 2 × 8 weeks of high-intensity interval training without (control period; CP) or with (IFP) intermittent fasting caloric restriction (20% reduction in weekly energy intake) on body composition and countermovement jump performance in active women. Body composition and CMJ was not different during the 1st compared to the 2nd pretest. During the CP, no change with HIIT was observed in body composition and CMJ. During the IFP fat mass decreased (*p* < 0.05) by 1.5 ± 0.2% corresponding to a decrease in fat mass of 1.0 ± 0.6 kg, and muscle mass increased (*p* < 0.05) by 2.0 ± 05% corresponding to 0.8 ± 03 kg of muscle mass (2.0%). The changes in fat and muscle mass during the IFP were greater (*p* < 0.015) than during the CP. CMJ performance improved during the IFP by 6.2 ± 1.2 cm, with no change during CP. In conclusion, intermittent fasting caloric restriction—combined with HIIT—can improve body composition and dynamic performance in active women.nutritiondietexercisetrainingHIIT

**Funding:** This research received no external funding

## 31. Cross Education is Modulated by Set Configuration

FariñasJuan^1^*GarcíaManuel Avelino Giráldez^1^MayoXian^2^CarballeiraEduardo^1^VázquezJessica Rial^1^SolerEliseo Igleisas^1^1Department of Physical Education and Sport, Faculty of Sports Sciences and Physical Education, University of A Coruña, Performance and Health Group, A Coruña, Spain2Observatory of Healthy and Active Living of Spain Active Foundation, Center for Sport Studies, King Juan Carlos University, Madrid, Spain*Correspondence: juan.farinhas@gmail.com; Tel.: +34 663-436-919The aim of this study was to compare the strength gains in nontrained limb after a unilateral strength training program with two different set configurations with the same repetition-to-rest ratio. Thirty-five participants were randomly assigned into three groups: Control (CON; n = 11), traditional (TT; n = 12) and cluster training groups (CT; n = 12). Experimental groups performed 10 sessions; TT performed 4 sets of 8 repetitions with the 10 RM load with three-min-rest between each set. CT completed 32 sets of one repetition with 17.5 s between each repetition. Before and after intervention, participants performed 1RM, 10RM, anthropometry (ANT); muscle thickness (MT) and pennation angle (PA) in vastus lateralis (VL), maximum isometric contraction (MVC), electromyography (EMG), M-wave and number of repetitions during postests with 10 RM pretest load (10RMr) tests. Regarding trained limb, increases in 1RM and 10RMr tests in both TT (*p* < 0.001) and CT (*p* = 0.001) were detected. No differences were obtained regarding MT, PA and root mean square analysis (RMS) of EMG. For nontrained limb, increases in TT regarding 1 RM (*p* < 0.001) and significant decreases in RMS (*p* = 0.041) in CT were detected. No changes were observed for 10RMr, MT, PA and M-Wave. We can conclude that cross education phenomenon takes place after most fatiguing protocols without structural changes.cross educationset configurationunilateral strength training

**Funding:** This research received no external funding.

## 32. Six Months of MultiComponent Training Improves Fitness Level in Frail or Pre-Frail Older People

Fernández-GarcíaÁngel Iván^1^,^2^,^3^Navarrete-VillanuevaDavid^1^,^2^,^3^,^4^MoradellAna^1^,^2^,^3^,^5^Gómez-BrutonAlejandro^1^,^2^,^3^,^5^CasajúsJosé Antonio^1^,^2^,^3^,^4^,^6^Gómez-CabelloAlba^1^,^2^,^3^,^5^,^6^,^7^Vicente-RodríguezGermán^1^,^2^,^3^,^5^,^6^*1GENUD (Growth, Exercise, NUtrition and Development) research group, University of Zaragoza, Zaragoza, Spain2Instituto Agroalimentario de Aragón -IA2- (CITA-University of Zaragoza), Zaragoza, Spain3Red Española de Investigación en Ejercicio Físico y Salud en Poblaciones Especiales (EXERNET), Zaragoza, Spain4Faculty of Health, Department of Physiatry and Nursing, University of Zaragoza, Zaragoza, Spain5Faculty of Health and Sport Science (FCSD, Ronda Misericordia 5, 22001-Huesca, Spain), Department of Physiatry and Nursing, University of Zaragoza, Zaragoza, Spain6Centro de Investigación Biomédica en Red de Fisiopatología de la Obesidad y Nutrición (CIBERObn)7Centro Universitario de la Defensa, Murcia, Spain*Correspondence: gervicen@unizar.esAging is associated with the impairment of functional capacity and exercise seems to be relevant to frailty prevention and treatment. The purpose of this study was to analyze the impact of a six-month multicomponent exercise program (MCEP) on the fitness performance of frail and pre-frail elderly. A total of 125 elderly with values of frailty or pre-frailty in the short physical performance battery participated in the study (34 men and 91 women, average age: 80.4 ± 6.0 years). The sample was divided into control (CG = 50) and intervention group (IG = 65). IG performed a MCEP of six months, three days per week. Fitness was assessed through the senior fitness test battery and the following additional tests: handgrip and timed-up and go with and without dual task. An ANOVA for repeated measures was completed to analyze the performance development along the six months and to compare the progress and interactions within and between groups. IG improved significantly in all assessed parameters (*p* < 0.05), while CG did not show significant changes in any fitness tests. Group by time interaction were found in all tests, except in handgrip, sit-and-reach and back scratch (*p* ≤ 0.01). A MCEP at six months showed positive effects on the physical fitness of frail and pre-frail elderly people.exerciseelderfrailty

**Funding:** This research was funded by the University of Zaragoza (UZ 2008-BIO-01), Centro Universitario de la Defensa de Zaragoza (UZCUD2017-BIO-01) and Ministerio de Economía, Industria y Competitividad (DEP2016-78309-R). Ángel Iván Fernández García received a Grant from de Government of Spain.

## 33. Does Maturity Status Influence Ranking, Anthropometry and Fitness Performance of Under-15 High-Level Tennis Players?

Fernández-GarcíaÁngel Iván^1^,^2^Vicente-RodríguezGermán^1^,^2^*Sanz-RivasDavid^3^,^4^del CosoJuan^5^Gallo-SalazarCésar^6^Fernández-FernándezJaime^7^1GENUD (Growth, Exercise, NUtrition and Development) research group, University of Zaragoza, Zaragoza, Spain2Faculty of Health and Sport Science, University of Zaragoza, Huesca, Spain3Faculty of Physical Activity and Sport Sciences, Camilo José Cela University, Madrid, Spain4Royal Spanish Tennis Federation (RFET), Madrid, Spain5Exercise Physiology Laboratory, Sport Sciences Institute, Camilo José Cela University, Madrid, Spain6Sport Sciences Institute, Camilo José Cela University, Madrid, Spain7Faculty of Physical Activity and Sports Sciences, University of León, León, Spain*Correspondence: gervicen@unizar.esFunctional capacities are related to biologic maturation. The purpose of this study was to analyze the influence of maturity status (average or late) in ranking, anthropometry and fitness of Under-15 high-level tennis players. A total of 36 male high-level tennis players from Spain (average age: 14.63 ± 0.32 years) underwent anthropometry measurements and fitness evaluations. Physical performance was assessed through the next test: handgrip, countermovement jump (CMJ), medicine ball throws, serve velocity, 5-, 10- and 20-m sprint, shuttle sprint to forehand and backhand sides and hit-and-turn test. Additionally, national ranking was registered and maturity status was obtained predicting years from the peak height velocity using height, sitting height, leg length, chronological age and their interactions. An ANOVA was performed to analyze the influence of maturity status in the studied variables. Average maturity players were taller, heavier and had better values of handgrip, CMJ with non-dominant leg and medicine ball throws from backhand side than late maturity players (*p* < 0.05). Moreover, they were faster in 5- and 20-m sprints (*p* < 0.01). However, no differences were obtained in the rest of variables, including ranking. Players who were advanced in biologic maturation had better values in almost of analyzed variables although this did not translate into better national ranking.maturationracket sportsperformance

**Funding:** This research received no external funding.

## 34. Comparison of Linear, Hyperbolic and Double-Hyperbolic Models to Assess the Force–Velocity Relationship in Multi-Joint Exercises

AlcazarJulian^1^,^2^Pareja-BlancoFernando^3^Rodriguez-LopezCarlos^1^,^2^Sanchez-ValdepeñasJuan^3^Navarro-CruzRoberto^1^,^2^Cornejo-DazaPedro J.^3^AraIgnacio^1^,^2^AlegreLuis M.^1^,^2^*1GENUD Toledo Research Group, Universidad de Castilla-La Mancha, Toledo, Spain2CIBER of Frailty and Healthy Aging (CIBERFES), Madrid, Spain3Physical Performance & Athletic Research Center, Universidad Pablo de Olavide, Seville, Spain*Correspondence: luis.alegre@uclm.comThis study assessed the validity of linear, hyperbolic and double-hyperbolic models to fit measured force–velocity (F–V) data in multi-joint exercises. Force-joint angle and F–V relationships were assessed in 10 cross-training athletes and 14 recreationally resistance-trained subjects in the unilateral leg press (LP) and bilateral bench press (BP) exercises, respectively. A force plate and a linear encoder were installed to register external force and velocity, respectively. Muscle excitation was assessed by surface EMG recording of the quadriceps femoris, biceps femoris and gluteus maximus muscles. Linear, Hill’s (hyperbolic) and Edman’s (double-hyperbolic) equations were fitted to the measured F–V data and compared. Measured F–V data were best fitted by double-hyperbolic models in both exercises (*p* < 0.05). F–V data deviated from the rectangular hyperbola above a breakpoint located at 90% of measured isometric force (*F*_0_) and from the linearity at ≤45% of *F*_0_ (both *p* < 0.05). No differences were found between muscle excitation levels below and above the breakpoint (*p* > 0.05). Associations between variables obtained from linear and double-hyperbolic models were noted for *F*_0_, maximum muscle power and velocity between 25–100% of *F*_0_ (r = 0.70 − 0.99; all *p* < 0.05). The F–V relationship in multi-joint exercises was double-hyperbolic. However, linear models may be valid between 25–100% of *F*_0_.torque-velocitymuscle powermuscle mechanics

**Funding:** This work was supported by the Ministerio de Economía y Competitividad of the Spanish Government (MINECO/FEDER, EU) under Grants DEP2015-69386-R and BES-2016-077199; Biomedical Research Networking Center on Frailty and Healthy Aging (CIBERFES) and FEDER funds from the European Union under Grant CB16/10/00477; and Ministerio de Educación, Cultura y Deporte of the Spanish Government under Grant FPU014/05106.

## 35. Six-Month Multicomponent Training Program Improves Muscle Strength in Frail and Prefrail Elderly People

Subías-PeriéJorge^1^,^2^,^3^,^4^,^5^Vicente-RodríguezGermán^1^,^2^,^3^,^4^,^6^Fernández-GarcíaÁngel Ivan^1^,^2^,^3^Moradell-FernándezAna^1^,^2^,^3^,^4^Navarrete-VillanuevaDavid^1^,^2^,^3^,^4^,^5^Marín-PuyaltoJorge^1^,^2^,^3^,^4^Gómez-CabelloAlba María^1^,^2^,^3^,^4^,^6^,^7^MallénJosé Antonio Casajús^1^,^2^,^3^,^5^,^6^*1GENUD (Growth, Exercise, NUtrition and Development) research group, University of Zaragoza, Zaragoza, Spain2Instituto Agroalimentario de Aragón -IA2- (CITA- University of Zaragoza), Zaragoza, Spain3Red española de Investigación en Ejercicio Físico y Salud en Poblaciones Especiales (EXERNET)4Faculty of Health and Sport Science (FCSD, Ronda Misericordia 5, 22001-Huesca, Spain), Department of Physiatry and Nursing, University of Zaragoza, Zaragoza, Spain5Faculty of Health, Department of Physiatry and Nursing, University of Zaragoza, Zaragoza, Spain6Centro de Investigación Biomédica en Red de Fisiopatología de la Obesidad y Nutrición (CIBERObn), Madrid, Spain7Centro Universitario de la Defensa, Murcia, Spain*Correspondence: joseant@unizar.esExercise seems to be relevant treatment to frailty prevention in elderly individuals. The aim of this study was to examine the effect of a six-month multicomponent training (MCT) program on the elbow flexion (EF), knee extension (KE) and shoulder abduction (SA) muscle strength. A total of 125 frail and prefrail participants enrolled in the study (average age: 80.4 ± 6.0 years). Sixty-eight seniors were conveniently included in the experimental group (EG) and performed an MCT program for six months, three sessions/week. The other 55 individuals were included in the control group (CG), which continued with their habitual routine. All subjects were assessed before and after intervention through Lafayette hand-held dynamometer. Maximum strength, average strength and time to maximum strength were recorded for each exercise. A repeated-measures ANOVA was used to compare the groups. After six months of MCT, the EG significantly improved maximum, average and time to maximum EF and KE muscle strength. CG only improved time to maximum SA muscle strength. Group by time interactions were found for maximum and average EF muscle strength and time to maximum SA muscle strength (all *p* < 0.05). The MCT can increase muscle strength, in frail and prefrail elderly people. Consequently, they may improve their functional capacity.exercisedynamometerhealth

**Funding:** The Elderly EXERNET Multicenter Study was supported by University of Zaragoza (UZ 2008-BIO-01), Centro Universitario de la Defensa de Zaragoza (UZCUD2017-BIO-01) and Ministerio de Economía, Industria y Competitividad (DEP2016-78309-R). Jorge Subías Perié received a Grant from de Government of Spain.

## 36. Effect of Motivation on Amateur Runners’ Resilience and Prevalence of Injury

León-GuereñoPatxi^1^*Tapia-SerranoMiguel Angel^2^CastañedaArkaitz^1^Aguayo-BenitoYolanda^3^Vaquero-SolísMikel^2^MiguelPedro Antonio Sánchez^2^1Faculty of Psychology and Education, University of Deusto, Donostia-San Sebastián, Spain; arkaitz.castaneda@deusto.es2Department of Didactics of Musical, Plastic and Body Expression, Faculty of Teaching Training, University of Extremadura 2, Cáceres, Spain; matapiese@unex.es (M.A.T.-S.); mivaquero@unex.es (M.V.S.); pesanchezm@unex.es (P.A.S.M.)3Gobierno Vasco/Eusko Jaurlaritza, Vitoria-Gasteiz, Spain; yaguayo@alumni.unav.es*Correspondence: Patxi.leon@deusto.es; Tel.: +34-6577701408(1) Background: The aim of this study was to test the association between resilience and the intrinsic and extrinsic motivation with the number of injuries in long-distance athletes; (2) Methods: Participants were 773 long-distance athletes, 556 men (age: 44.09 ± 9.35) and 217 women (age: 40.66 ± 9.55). The number of injuries were examined with a retrospective survey. The intrinsic and extrinsic motivation were evaluated with the behavioral regulation in exercise questionnaire (BREQ-3) and the resilience with the Spanish version of CD-RISC; (3) Results: Results showed that the resilience level and the intrinsic and extrinsic motivation were related with the number of injuries after the adjusted of the covariables study: age and sex. The results of Model 1 showed a significant association between the resilience level and the number of injuries (*p* < 0.05). Model 2 did not reveal significant association between intrinsic motivation and the number injuries (*p* > 0.05); (4) Conclusions: Therefore, it could be said that athlete resilience abilities help to explain the number of injuries in amateur runners, while effects of motivational characteristics remain unclear.intrinsic motivationextrinsic motivationresilience

**Funding:** This research received no external funding.

## 37. Effect of Motivation on Perceived Health Status among Long-Distance Runners

León-GuereñoPatxi^1^*Tapia-SerranoMiguel Angel^2^CastañedaArkaitz^1^Aguayo-BenitoYolanda^3^Vaquero-SolísMikel^2^MiguelPedro Antonio Sánchez^2^1Faculty of Psychology and Education, University of Deusto, Donostia-San Sebastián, Spain; arkaitz.castaneda@deusto.es2Department of Didactics of Musical, Plastic and Body Expression, Faculty of Teaching Training, University of Extremadura 2, Cáceres, Spain; matapiese@unex.es (M.A.T.-S.); mivaquero@unex.es (M.V.-S.); pesanchezm@unex.es (P.A.S.M.)3Gobierno Vasco/ Eusko Jaurlaritza, Vitoria-Gasteiz, Spain; yaguayo@alumni.unav.es*Correspondence: Patxi.leon@deusto.es; Tel.: +34-6577701408(1) Background: The number of athletes taking part in different running events has increased significantly in the last decade. The aim of this study was to test significant differences between BMI and the intrinsic and extrinsic motivation depending on the sex; (2) Methods: The sample was formed by 749 long-distance athletes, 540 men (age: 44.09 ± 9.35; BMI: 23.99 ± 2.36) and 209 women (age: 40.66 ± 9.55; BMI: 21.16 ± 2.23). BMI was calculated as the ratio of weight (in kg)/squared standing height (m^2^). The intrinsic and extrinsic motivation were evaluated with the behavioral regulation in exercise questionnaire (BREQ-3); (3) Results: We found a significant association between BMI and sex (*p* < 0.05). Regarding the motivation, the results were significant for intrinsic (*p* < 0.05) and extrinsic (*p* < 0.05) motivation respect to sex; (4) Conclusions: It could be said that runner BMI is positively associated with motivational characteristics—which are also statistically different with gender. Following these findings, we can see the important effect of intrinsic and extrinsic motivation on runner health status, needing to be treated differently for sportswomen and sportsmen.runningintrinsic motivationextrinsic motivation

**Funding:** This research received no external funding.

## 38. Effect of Resistance Training and Gluten-Free Diet on Body Composition and Strength in Celiac Women

Martínez-RodríguezAlejandro^1^*Loaiza-MartínezDaniela A.^2^Marcos-PardoPablo J.^2^Sánchez-SánchezJavier^3^AlacidFernando^4^Prats-MoyaSoledad^1^Rubio-AriasJacobo A.^5^1Department of Analytical Chemistry, Nutrition and Food Science, Faculty of Sciences, University of Alicante, Ctra. San Vicente del Raspeig, s/n, 03690 Alicante, Spain; maria.prats@ua.es2San Antonio Catholic University of Murcia, Murcia, Spain; daloaiza@alu.ucam.edu (D.A.L.-M); pmarcos@ucam.edu (P.J.M.-P.)3European University of Madrid, Madrid, Spain; javiersanchezsanchez22@gmail.com4University of Almería, Almería, Spain; falacid@ual.es5Polytechnic University of Madrid, Madrid, Spain; ja.rubio@upm.es*Correspondence: amartinezrodriguez@ua.esCeliac disease is an immunological disorder that generates an inflammatory process in response to the presence of gluten. Because of this, patients who do not receive enough nutrients cannot maintain their muscle and body mass. The aim of this study was to compare the handgrip strength and body mass index of celiac women before and after an intervention of three months of physical activity and a gluten-free diet. Twenty-one celiac women over 55 years old participated in the study. The sample were divided randomly into three different groups (n = seven in each group): gluten-free diet + resistance training (DT); gluten-free diet, but no physical activity (DNT); and control group. DT group conducted resistance training two hours per week during a three-month period. Body composition was evaluated by digital weight scale and stadiometer following International Society for the Advancement of kinanthropometry guidelines. Body mass index (BMI) was calculated as weight (kg)/ standing height (m)^2^. DT group decrease their BMI and increase their strength, but the group DNT maintain their BMI and maintain their strength and control group maintain their BMI but decrease their strength. Resistance training and gluten-free diet showed an improvement of body composition and strength in adult-older celiac women.nutritionexercisetraining

**Funding:** This research was funded by Generalitat Valenciana (Concelleria D’ Educació, Investigació Cultura I Esport), Grant Number GV/2017/112.

## 39. Does a 200-m Sprint Cause the Onset of Inspiratory Muscle Fatigue?

PuchMiguel^1^*LópezMaría José^2^*EzequielAida^1^1Sport Science and Physical Activity Department, Pablo Olavide University, 41013 Seville, Spain; aezerod@alu.upo.es2Psychology Department, National University of Distance Education, 41007 Seville, Spain*Correspondence: mapucgar@alu.upo.es (M.P.); mlopez6142@alumno.uned.es (M.J.L.)Numerous research studies have found inspiratory muscle fatigue (IMF) following the performance of various types of foot races. However, no study has analyzed whether IMF 1 is triggered by the 200-meter sprint. Fourteen physically active subjects performed a standardized warm-up to minimize heterogeneity in the effects of performing different warm-up protocols, and subsequently executed the 200 m test on an athletics tartan. Both before the warm-up and after the 200 m test, each participatory inspiratory muscle strength (IMS) was evaluated through a maximum voluntary inspiratory pressure in mouth (MVIP_m_) test. The percentage of decrease in MVIP_m_ between the pretest and the posttest was considered representative of the IMF associated with the 200 m sprint test. Participants presented a significantly lower MVIP_m_ after the 200 m sprint (M = 1.492, SD = 0.35, SE = 0.09, t(13) = 3.31, *p* ˂ 0.01, r = 0.68) than before (M = 1.635, SD = 0.35, SE = 0.09), consequently defining IMF corresponding to 8.8% ± 9.5%. This research shows that, after sprinting 200 m, temporary incapacity occurs in the production of inspiratory muscle strength.short-duration racesdiaphragmrespiratory muscles

**Funding:** This research received no external funding.

## 40. Neither Asymmetry of Maximal Force Nor Asymmetry of Force Variability of Plantar Flexor Muscles Appear to Influence Elderly Postural Control

SilvaNilson R. S.*BarbosaRoberto N.GomesMatheus M.Laboratory of Biomechanics and Motor Control, School of Physical Education and Sport of Ribeirão Preto, University of São Paulo, São Paulo, Brazil*Correspondence: nilsonribeiro@usp.brBackground: This study analyzed the relationship between symmetry of the force variability of the plantar flexor muscles (PMF) with postural control in older women. Methods: Twenty participants were recruited for the study. Data of postural control were acquired by a force platform, which provided the mean amplitude (MA) and the mean velocity (MV) of the center of pressure dislocations and area 95% (A95). An experimental device with two coupled load cells was developed to measure force variability (FV) and maximal force (MF). In this experiment, to determine FV, the volunteers remained seated with parallel feet and performed MF attempts or maintained isometric plantar flexion force for 20 s, at relative intensities of 5% and 10% of the MF. Asymmetry indices were calculated from the dominant leg variability (DLV) and the non-dominant leg variability (NDLV) with the following equation: AFV = [NDLV-DLV/0.5*(NDLV + DLV)] × 100. Results: Pearson’s correlation test did not indicate significant associations between the asymmetries on both intensities (5% and 10% of the MF) and the center of pressure displacement variables Conclusion: Postural control of older adults is not associated with the symmetry of the force variability and with the symmetry of maximal force of the PFM.body swayforce fluctuationaging

**Funding:** This research was funded by the São Paulo Research Foundation, grant number 2017/05174-8.

## 41. Load–Velocity Relationship of Full-Squat Exercise in U-15 Soccer Players

García-GonzálezP.*VelascoJ. A.University Pablo de Olavide, Sevilla, Spain; joseavel2@hotmail.com*Correspondence: pgargon5@alu.upo.esMaximum repetition (1RM) has been used as the main control parameter in resistance training for years. However, recent studies have proposed the movement velocity as a practical and useful alternative. The purpose of this study was to analyze the relationship between the mean propulsive velocity (VMP), and the relative load expressed as a percentage of the maximum repetition (%1RM) in U-15 soccer players. Nine young male soccer players performed a progressive test until reaching their maximum repetition in the full squat exercise. Bar velocity was measured by a linear velocity transducer. A strong relationship between VMP and %1RM was found (R^2^ = 0.96). Prediction equations to estimate load from velocity were obtained. Velocity values for the 1RM are considerably higher than in bench press exercise and slightly higher than in pull-up exercise. VMP values obtained for each %1RM are lower than other values obtained in previous studies which focused on the full squat exercise. Because of these subjects are slower, it is recommended to use the equations provided in this study for the full squat exercise training in U-15 soccer players.resistance trainingmuscle strengthathletic performancevelocity-based trainingbar velocity

**Funding:** This research received no external funding.

## 42. Can the Likelihood of Rugby Match Injuries Amongst Male Adolescent Players Be Predicted Using Basic Psychological Needs Survey Scores?

McKeownPaul*PricePhilCleatherDanielFaculty of Sport, Health & Science, St Mary’s University, Waldegrave Road, Strawberry Hill, Twickenham, TW1 4SX, UK; phil.price@stmarys.ac.uk (P.P.); daniel.cleather@stmarys.ac.uk (D.C.)*Correspondence: pmckeown@coachingperformance.co.ukIndividuals who are unsatisfied in terms of their basic psychological needs of autonomy, competence and relatedness are considered more likely to suffer from unwanted stress. Unwanted stress has been observed to increase the likelihood of sporting injury among adolescents. To further examine these concepts, 121 rugby union players aged between 14–18 years were asked to complete a basic psychological needs satisfaction in general survey 2 days prior to playing competitive rugby matches. A Mann–Whitney U test showed that players who received injuries during their games were significantly more likely to have recorded lower relatedness survey scores than the uninjured players (μinj = 41.56 ± 5.61 and μuninj = 46.24 ± 5.47, respectively, U = 237, *p* < 0.01). To better understand what particular relatedness factors were affecting this population, a deviant case analysis was conducted with a range of high and low relatedness scoring participants. The interviewees highlighted how perceived levels of team cohesion and social support could affect a player’s feelings of relatedness and their likelihood of incurring match injuries. These findings could help justify the use of BPN surveys within schools and rugby clubs as an inexpensive method to identify individuals who report low levels of relatedness satisfaction and intervene to help avoid the associated elevated risk of injury.keywordrugbyadolescentbasicpsychologicalneedsinjurywellbeingschoolswellnessteam

**Funding:** This research received no external funding.

## 43. Does Resisted Sled Sprinting Improve the Physical Qualities of Elite Youth Soccer Players of Differing Maturity Status?

MorrisRhys^1^*MyersTony^2^EmmondsStacey^3^SingletonDave^4^TillKein^3^1Coventry University, Conventry, UK2Newman University; Birmingham, UK3Leeds Beckett University, Leeds, UK4Birmingham City FC, Birmingham, UK*Correspondence: ac9669@coventry.ac.ukSled towing has been shown to be an effective tool to enhance the physical qualities in youth soccer players. The aim of this study was to evaluate the impact of a six-week sled towing intervention on muscular strength, speed and power in elite youth soccer players of differing maturity status’ (pre-, circa-, post-PHV). Method: A total of 73 male elite youth soccer players aged 12–18 years (pre-PHV n = 25; circa-PHV n = 24; post-PHV n = 24) were recruited for this study from professional soccer academy. Data collection consisted of anthropometric and physical qualities which were collected at the start of the intervention (T1) and at the end of the six-week intervention (T2). Physical qualities were assessed using 10- and 30-m sprint speed, countermovement jump for lower limb power and isometric mid-thigh pull for lower limb strength. The training intervention consisted of six weeks of resisted sled towing (FH Pro Mini Speed Sledge Team Series), two sessions per week with a total of 12 sessions during the competitive season. Each session consisted of 10 sprints over 20-m distance. To model pairwise differences between pre and post-scores within each maturation group, Bayesian regression models were fitted using a Student-t distribution (Haff, & Aas, 2008). Probability values of a change being greater than 0 (*p* > 0 or <0) were provided with a standardized effect size calculated from the posterior estimates and again the uncertainty illustrated with lower and upper 95% HDIs. Results: There were no significant changes in pre- to post-performance measures following the six-week intervention for any of the maturation groups. It appears that loadings of 10–30% BM do not induce performance changes in elite youth soccer players over a range of performance measures.youth developmentsled towingmaturationLTADsoccerstrength developmentspeed developmentIMTP

**Funding:** This research received no external funding.

## 44. Evolution of the Velocity Loss and the Glycolytic Involvement throughout Two Resistance-Training Programs Differing in set Configuration

Rial-VázquezJessica*Iglesias-SolerEliseoFariñasJuanRúa-AlonsoMaríaPerformance and Health Group (Ph-g), Department of Physical and Sports Education, University of A Coruña, A Coruña, Spain*Correspondence: Jessica.rial@udc.esThe main aim of this study was to contrast the evolution of velocity loss and the glycolytic involvement throughout two five-week training programs differing in set configuration. Thirty-nine subjects were recruited and assigned to three groups: Traditional, cluster and control. A total of 10 training sessions were carried out by the experimental groups in which traditional training group performed 4 sets of 8 repetitions with 5 min of recovery, and cluster training group performed 16 sets of two repetitions with one min of rest between sets. Four exercises were performed: bench, press, parallel squat, lat pull-down and leg curl) and pause between exercises was 5 min. Capillary blood lactate concentration was measured at baseline, one and three min after session 1, 5 and 10 and two ratios (last to the first repetitions ratio and mean to maximum ratio) were calculated in order to examine the velocity loss for bench press and parallel squat exercise. Greater velocity loss and lactate concentration was observed for cluster group in comparison with traditional throughout the 10 training sessions.cluster traininglactatefatiguestrength

**Funding:** This research received no external funding.

## 45. Effects of Reverse vs. Undulating Periodization with the Same Mean Relative Intensity on Squat Gains

Riscart-LópezJavier*Pareja-BlancoFernandoLeón-PradosJuan AntonioPhysical Performance & Athletic Research Center, Faculty of Sports Science, Pablo de Olavide University, Ctra. Utrera km 1, 41013 Seville, Spain; fparbla@gmail.com (F.P.-B.); jleopra@upo.es (J.A.L.-P.)*Correspondence: javiriscart@gmail.com; Tel.: +34-671-17-78-95This aim of this study was to compare the effects at different regions of the load–velocity relationship on squat gains of two velocity-based resistance-training (VBRT) programs with the same mean relative intensity (65% 1RM), using the type of periodization (reverse periodization (RP) vs. undulating periodization (UP)) as the independent variable. Twenty-two young males were randomly assigned to a RP (n = 12) or UP (n = 10) group. Each group followed an eight-week VBRT program using the full squat exercise, while movement velocity was monitored for every repetition, using a linear velocity transducer. Pre- and post-training assessments included: 1RM squat strength, average mean propulsive velocity (MPV) attained against all absolute loads common to pre and post (AV), average MPV attained against absolute loads common to pre and post that were moved faster than one m/s (AV > one, ‘light’ loads) and average MPV attained against absolute loads common to pre and posttests that were moved slower than one m/s (AV < one, ‘heavy’ loads). Significant time × group interactions in favor of RP were observed for AV (*p* < 0.05) and AV > one (*p* < 0.05). Both groups attained significant 1RM strength gains (*p* < 0.001), AV (*p* < 0.001), AV > one (*p* < 0.001–0.05) and AV < one (*p* < 0.001) for RP and UP groups, respectively. These results showed that RP induce similar or greater effects at different regions of the load–velocity relationship on squat gains than UP.dose–responsestrengthtraining

**Funding:** This research received no external funding.

## 46. Similar Velocity Loss between Men and Women during Resistance Training Sessions Differing in Set Configuration

Rúa-AlonsoMaría^1^*Iglesias-SolerEliseo^1^MayoXian^1^,^2^Rial-VázquezJessica^1^FariñasJuan^1^1University of A Coruna, Performance and Health Group, Department of Physical Education and Sport, Faculty of Sports Sciences and Physical Education, 15179A Coruña, Spain2Active and Healthy Lifestyle Observatory, Centre for Sport Studies, King Juan Carlos University, 28942 Madrid, Spain*Correspondence: maria.rua@udc.esThe anato-physiological sex differences are responsible for the dissimilarities in neuromuscular performance and fatigability. The aim was to analyze the differences between men and women regarding fatigability in two resistance-training (RT) sessions differing in set configuration. A total of 41 sport science students (29 males and 12 females) performed two RT sessions (knee extension (KE), leg curl, lat pull, bench press (BP) and parallel squat (SQ)) equated by intensity, volume and total rest and differed by set configuration: four sets of 10 repetitions with two min rest between sets (traditional sets: TS) and eight sets of five repetitions with 51 s rest between sets (cluster sets: CS). During sessions, mean propulsive velocity (MPV) of every repetition was recorded and averaged for KE, BP and SQ. Moreover, the overall maintenance of velocity (MMR) and the velocity (5LFR) were calculated. Analyses reported a main effect of session for MVP, MMR and 5LFR (*p* < 0.001–0.017) in KE and BP, where CS had higher values of both parameters than TS. Additionally, main effect of sex was detected for MPV in KE (*p* < 0.001). Summarily, CS produces less neuromuscular fatigue than TS in some exercises during a whole-body multi-exercise RT but set configuration does not modulate the sex-related differences regarding the fatigability.fatigabilitysex differencescluster training

**Funding:** This research received no external funding.

## 47. Effect of Training Program on Mixed Contractions on both the Maximum Force and Explosive Force of the Lower Limbs: A Study of Football Players under the Age of 17 Years in Algeria

HouariSaidia^1^*HamidNahal^2^HakimDani^3^1Director of the Laboratory LABOMES, University Center of Tissemsilt, Tissemsilt, Algeria2United Head of Research Unit, University Center Tissemsilt, Tissemsilt, Algeria; Hamid-1432@hotmail.com3Bouira university, Bouria, Algeria,dannyniohakim@gmail.com*Correspondence: drsaidia@hotmail.com; Tel.: +0021346551032The aim of the present research was to propose a training program (mixed contractions) to develop maximum power and explosive power levels of the lower limbs of football players of the State University of Tiaret under the age of 17 years. We also sought to experiment with some tests for the evaluation and development both maximum strength and strength. This study was conducted in the period from 10 October 2016 to 15 March 2017. Studies were conducted on the Olympic football team under the age of 17 years for the 2016/2017 sports season. Members of the 25-person research sample were under the age of 17 years. Five players of the basic research sample were included from a pilot study. The remaining 20 players applied to the proposed program. Two players sustained injury, which left the program with 18 players after the parity process, i.e., the selection of tests suitable for the explosive power of the maximum force. After proposing the tests to the group, the arbitrators choose an appropriate match for intended development using the application of the proposed training program (mixed contractions). A total of two training courses per week (Saturday and Wednesday), the size of a courier was estimated to one hour for the training courses for six weeks. After the tests were conducted, the results and then analyzed by appropriate statistical means.proposed trainingmixed contractionexplosive forcemaximum force

**Funding:** This research received no external funding.

## 48. Acute effect of Resistance Exercises on Heart-Rate Variability: A Meta-Analysis

UdayangaSajith M. A.^1^*Rubio-AriasJacobo A.^1^AlcarazPedro E.^1^,^2^ChungLinda H.^1^,^2^1Faculty of Sport Sciences, Catholic University of Murcia, UCAM, 30107 Murcia, Spain2UCAM Research Center for High Performance Sport, Catholic University of Murcia, UCAM, 30107 Murcia, Spain*Correspondence: sumarasingha@alu.ucam.eduThe purpose of this meta-analysis was to study the acute effect of resistance exercise (ARE) on heart rate variability (HRV) parameters and identify what are the ARE-related moderating factors on HRV. Searches were conducted in PubMed-Medline, Web of Science, SPORTDiscus and Cochrane Library (12 May 2019). PRISMA declaration was followed. Categorical variables like training intensity and gender were assessed using subgroup analysis. Meta-regression analyses were conducted for continues variables (number of repetitions, sets, rest between sets, exercises per workout, volume and load). A total of 26 studies met the inclusion criteria. The main effects analyzed between pre- and posttest interventions demonstrated that there was a significant increase in LFnu (*p* < 0.01) and LF/HF (*p* < 0.01) and a significant decrease in RMSSD (*p* < 0.01), HFnu (*p* < 0.01) and TP (*p* < 0.01) after ARE. A subgroup analysis showed that gender (*p* = 0.009) is a moderating factor of TP. The meta-regression analysis revealed that training load (*p* = 0.01) is a moderating factor of RMSSD. This meta-analysis demonstrates that there is a withdrawal of parasympathetic modulation and activation of sympathetic modulation following ARE (~post-30 min) among young healthy or resistance trained adults. Furthermore, training load can be considered as a moderating factor of RMSSD while gender plays a role for changes in TP following ARE.sympatheticparasympatheticresistance exerciseacute effectHRV

**Funding:** This research received no external funding.

## 49. Body Composition Changes in Frail and Prefrail Older Adults after Six Months of a Multicomponent Exercise Program: Preliminary Results from the EXERNET-Elder 3.0 Project

MoradellAna^1^,^2^,^3^,^4^*Fernández-GarcíaAngel Iván^1^,^2^,^3^Navarrete-VillanuevaDavid^1^,^2^,^3^,^4^,^5^Subías-PiereJorge^1^,^2^,^3^,^4^,^5^SagarraLucía^6^Lozano-BlesaGabriel^1^,^2^,^3^,^4^CasajusJose Antonio^1^,^2^,^3^,^5^,^7^Vicente-RodríguezGermán^1^,^2^,^3^,^4^,^7^Gómez-CabelloAlba^1^,^2^,^3^,^4^,^7^,^8^1GENUD (Growth, Exercise, Nutrition and Development) research group, Universidad de Zaragoza, Zaragoza, Spain2Instituto Agroalimentario de Aragón -IA2- (CITA-Universidad de Zaragoza), Zaragoza, Spain3Red española de Investigación en Ejercicio Físico y Salud en Poblaciones Especiales (EXERNET)4Faculty of Health and Sport Science (FCSD, Ronda Misericordia 5, 22001-Huesca, Spain) Department of Physiatry and Nursing, University of Zaragoza, Zaragoza, Spain5Faculty of Health, Department of Physiatry and Nursing, University of Zaragoza, Zaragoza, Spain6Grupo VALORA, Universidad San Jorge, Zaragoza, Spain7Centro de Investigación Biomédica en Red de Fisiopatología de la Obesidad y Nutrición (CIBERObn)8Centro Universitario de la Defensa, Zaragoza, Spain*Correspondence: amoradell@unizar.esThe aim of this study was to evaluate changes on body composition of 129 frail and prefrail older adults (80.4 ± 5.9 years) after a six-month multicomponent exercise program. A control group (CON) (n = 62) and training group TRAIN (n = 63), were evaluated before and after the intervention period. Weight and height were measured to calculate BMI. A body composition analyzer was used to estimate fat mass (FM) and total lean mass (TLM) both in kg. Relative skeletal muscle mass (kg) (RMM) described by Janssen et al. and fat mass percentage (FM%) were calculated. A *t*-Student’s test was performed to evaluate differences between groups before the intervention and ANOVA for repeated measures in order to compare differences before and after the six months within and between groups. No differences between groups nor group by time interaction were found before the intervention in any variable (all *p* > 0.05). Only increases in TLM and RMM and a reduction of the FM% were observed within the TRAIN group (TLM 45.6 ± 8.0 kg vs. 46.3 ± 8.3 kg; RMM 17.3 ± 4.7 kg vs. 17.72 ± 4.8 kg and FM% 37.4 ± 6.4% vs. 36.5 ± 6.4; pre- and post-evaluation, respectively). In conclusion, as changes are small, studies should study which factors are associated with bigger changes among these participants and the optimal doses of this exercise.frailtyeldermulticomponent trainingbody composition

**Funding:** The Elderly EXERNET Multicenter Study was supported by University of Zaragoza (UZ 2008-BIO-01), Centro Universitario de la Defensa de Zaragoza (UZCUD2017-BIO-01), Ministerio de Economía, Industria y Competitividad (DEP2016-78309-R) and Fondos FEDER. AMF received a Grant DGA 2017/2018. The authors are also grateful to all the collaborators and volunteers whose cooperation and dedication made this study possible.

## 50. Acute Effect of Transcranial Direct Current Stimulation on Running and Cycling Performance. A Systematic Review and Meta-Analysis

FernandoShyamali Kaushalya^1^*Romero-ArenasSalvador^1^Colomer-PovedaDavid^1^García-RamosAmador^2^MárquezGonzalo^1^1Department of Physical Education and Sports Science, Catholic University of Murcia, UCAM, 30107 Murcia, Spain; sromero@ucam.edu (S.R.-A); dcolomer@ucam.edu (D.C.-P.); gmarques@ucam.edu (G.M.)2Faculty of Education, Catholic University of Santísima Conception, Conception, Chile; amagr@ugr.es*Correspondence: skfernando@alu.ucam.edu; Tel.: +34-631207902Endurance tasks involving cycling or running time to exhaustion (TTE) or submaximal isometric tasks to volitional failure, spinal excitability decline as the motoneurons become progressively resistant to activation and the contractile capacity of the muscle fibers is reduced. Transcranial direct current stimulation (tDCS) is a technique that can transiently modulate the activity of a targeted brain area, and consequently, change exercise performance. The purpose of the present systematic review was to analyze the effect of anodal-tDCS on cycling or running TTE, time trial (TT) and sprint performance. We performed systematic literature in the Medline, SPORTDiscus and Science Direct databases. We included only randomized controlled trials with healthy people (18–50 years), in which a tDCS protocol was applied before cycling or running task. A sub-group analysis revealed a positive effect of a-tDCS on TTE (SMD = 0.32; 90% CI = 0.08, 0.56; *p* = 0.03), but not on TT (*p* = 0.95) or sprint (*p* = 0.43) performance. Enhancement in TTE performance could be related to a rise in cortical excitability, which increases output during exercise—thus reducing the supraspinal fatigue. Our results indicated that following a-tDCS can improve cycling and running performance. Contradictory results among studies may be related to the tDCS setup.tDCStime to task failureendurance

**Funding:** This research received no external funding.

## 51. Balance Exercise Improves Postural Sway, but Not Visual Feedback Regulation of Body Position

ĽubicaBöhmerová*JanaLuptákováPeterSchickhoferSabínaŠálováDušanHamar*Faculty of Sport and Physical Education, Comenius University Bratislava, Bratislava, Slovakia; jana.luptakova@uniba.sk (L.J.); sab.salova@gmail.com (S.S.)*Correspondence: lubica.bohmerova@uniba.sk (B.L.); dusan.hamar@gmail.com (H.D)This paper deals with the effect of exercise on an unstable surface on postural sway and visual feedback regulation of body position. A group of eight floorball players (mean age 17.0 ± 1.1 years, body height 176.7 ± 9.5 cm and weight 68.7 ± 7.4 kg) underwent a six-week training program consisting of exercises using fit-ball, BOSU-balance board and wobble board performed for 30 min twice per week. The control group consisted of eight male floorball players (mean age 18.0 ± 0.9 years, height 179.2 ± 4.4 cm, weight 73.0 ± 10.6 kg). Postural sway was assessed by means of the spring-based stabilographic system FiTRO angle sway check, which registers and samples the center of pressure (COP) at the rate of 100 Hz. The mean velocity of COP from 30 s stands was employed as a parameter of postural sway with and without visual control. Visual feedback regulation of body position with visual control was evaluated by means of FiTRO sway check. Subjects must performed corrective movements of the body in a horizontal plane in order to keep the COP displayed on the screen as close as possible to the flowing curve. As a parameter of visual feedback regulation of body position, a mean distance from COP to the flowing curve during the 30-s test was employed. Results showed that in contrast to the control subjects, the experimental group improved significantly postural sway under stable conditions, eyes open (from 19.2 ± 10.0 mm.s^−1^ to 13.9 ± 4.9 mm.s−1 and closed eyes 38.7 ± 16.4 mm.s−1 to 30.1 ± 12.7 mm.s−1). The index of feedback regulation of body position has changed only nonsignificantly, from 38.9 ± 3.0 to 39.6 ± 2.6 mm. It can be concluded that a six-week balance training improves postural sway, however, does not significantly affect visual feedback regulation of body position.balance abilitiespostural swaybalance trainingfloorballspring-based stabilographic platform

**Funding:** Supported by VEGA Grant No.: 1/0907/17.

## 52. How Postactivation Performance Enhancements Affect Semi-Tethered Swimming Kinetics

Cuenca-FernándezFrancisco*Ruiz-NavarroJesús JuanArellanoRaúlAquatics Lab, Department of Physical Education and Sports, Faculty of Sport Sciences, University of Granada, Granada, Spain*Correspondence: cuenca@ugr.es; Tel.: +34-627320795The aim of this study was to test if muscular performance is elevated in semi-tethered swimming tests by using a specific dry-land warm-up based on post-activation performance enhancements (denoted PAPE). Ten competitive swimmers conducted two tests to compare the effects of a PAPE warm up on an arm-stroke dry-land test and on a semi-tethered swimming test. Statistical differences between the variables collected through an adapted linear encoder were determined using a paired Student’s *t*-test. The experimental warm-up caused a positive effect on the dry-land test and a detrimental effect on the swimming test. Although improvements in performance were registered in dry-land conditions as a consequence of PAPE effect, these potentiated effects were not transferred into the water—the swimming time was longer (*p* = 0.014), the distance covered was shorter (*p* = 0.001) and the swimming power and velocity was lower (*p* = 0.001). Surprisingly—although the semi-tethered swimming procedure used in this study was not designed as a conditioning exercise to induce PAPE, but as a testing tool to assess the variations on performance as a consequence of it—the analysis of the effects obtained after this kind of protocol suggest a positive influence for stimulating maximal swimming efforts.warm-upstrength developmentperformance assessment

**Funding:** This research was funded by the Ministry of Economy, Industry and Competitiveness (Spanish Agency of Research) and the European Regional Development Fund (ERDF), grant number: DEP2014-59707-9 “SWIM: Specific Water Innovative Measurements applied to the development of International Swimmers in Short Swimming Events” and PGC2018-102116-B-I00 “SWIM II: Specific Water Innovative Measurements: applied to the improvement in performance.

## 53. Role of Change in Variables that Influence Physical and Technical Outcomes in Small-Sided-Games

HauerRichard^1^*TessitoreAntonio^2^TschanHarald^1^1University of Vienna, Vienna, Austria; harald.tschan@univie.ac.at2University of Rome “Foro Italico”, Rome, Italy; antonio.tessitore@uniroma4.it*Correspondence: richard.hauer@univie.ac.at; Tel.: +43-1-4277-48842(1) Small-sided games (SSGs) are a popular tool used in team sports. Nevertheless, uncertainty about the influence of different variables on the outcomes still exist. Therefore, the aim of this study was to investigate changes in bout-durations (BD) and rule-modifications (RM) on performance-parameters in SSGs; (2) Methods: First, differences between continuous (SSG-C) and intermittent (SSG-I) regimes and second, the influence of BD and RM were observed. Data were collected from 12 male lacrosse players. Interventions consisted of eight SSG-sessions. Players’ heart-rate (HR)max, endurance performance, mean %HRmax, time spent in 4 HR zones (HRz), rating of perceived exertion (RPE), and technical actions (TA) were collected. Statistical significance was set at *p* ≤ 0.05; (3) Results: Both SSG-regimes improved endurance performance (*p* = 0.00). Furthermore, SSG-C showed higher %HRmax (*p* = 0.00) and time spent in HRz4 (*p* = 0.00) compared to SSG-I. Noncontact regimes (NCR) showed lower RPE (p = 0.01) values and time spent in HRz2 (p = 0.04) and 3 (*p* = 0.05). Moreover, higher number of total events (*p* = 0.00), passes (*p* = 0.00) and shots (*p* = 0.04) were observed. Overall, our results support the hypothesis that RM influence SSG outcomes. According to these findings, we recommend implementing different BD and RM in SSGs to change the outcomes of such training in accordance with set goals.team sports trainingheart rate monitoringlacrosse

**Funding:** This research received no external funding.

## 54. Acute Mechanical and Metabolic Responses to Different Loading Conditions During Resisted Sprint Training

Bachero-MenaBeatriz^1^*Sánchez-MorenoMiguel^1^Pareja-BlancoFernando^2^Sañudo-CorralesBorja^1^1Department of Physical Education and Sport, University of Seville, Sevilla, Spain2Physical Performance & Sports Research Center, Department of Sports, Universidad Pablo de Olavide, Seville, Spain*Correspondence: beatriz.bachero@hotmail.comDespite scientific attention received by sled towing, there is still controversy over the loads that should be used to maximize gains in sprint performance. This study analyzed the acute mechanical and metabolic responses to resisted sprint training with five different loading conditions (0%, 20%, 40%, 60% and 80% body mass). Fifteen male subjects performed 8 × 20 m sprints with two min rest between each sprint with five different loading conditions. Subjects performed a battery of tests: lactate concentration, countermovement jump (CMJ), 20 m sprint (T20 m) and isokinetic knee extension and flexion contractions; at two different time-points: pre-exercise (PRE) and post-exercise (POST). Results revealed significant increases in blood lactate for all loading conditions, however, as sled loadings increased, higher blood lactate concentrations and increments in sprint times during training session were observed. Concerning the mechanical response, significant decreases in CMJ height from PRE to POST were found for all loading conditions. In addition, significant decreases in T20 m performance from PRE to POST were observed for 0% (*P* = 0.05) and 80% (*P* = 0.02). No significant differences from PRE to POST were observed for the isokinetic knee extension and flexion contractions. The loss of performance induced during training and the blood lactate concentration were significantly higher as the load increased, indicating that the higher is the load employed during resisted sprint training, the higher is the mechanical and metabolic fatigue produced. On the other hand, concerning the mechanical responses, fatigue in CMJ after the training protocol was similar for all loading conditions. Still, 20-m sprint performance was negatively affected only for 0% and 80%, likely due to different mechanisms: the increase in T20 m for 0% was due to a significant increase in the maximum velocity phase T1020 m, whereas the 80% load did not increase significantly in that phase.sled towingrunningfatiguelactatemuscle damage

**Funding:** Please add: This research received no external funding.

## 55. Assessment of the Polyunsaturated Fatty Acids (PUFA) Omega 3/Omega 6 in Erythrocyte Membrane in Professional Soccer Players

MichalczykMałgorzata Magdalena*GawełczykMateuszZającAdamInstitute of Sport Sciences, The Jerzy Kukuczka Academy of Physical Education, Katowice, Poland; m.gawelczyk@awf.katowice.pl (M.G.); a.zajac@awf.katowice.pl (A.Z.)*Correspondence: m.michalczyk@awf.katowice.plBackground: The aim of this study was a quantitative assessment of fatty acids in erythrocyte membrane in professional soccer players. PUFA, MUFA, SFA and TRANS levels in erythrocyte membranes are a reflection of the content of these acids in the diet. Methods: Forty males, professional soccer players (age 25.05 ± 4.5 y; height 182.5 ± 4.9 cm; body mass 78 ± 5.5 kg) took part in the study. Evaluation of % PUFA (omega3-EPA, DHA/omega-6-LA,AA)/monounsaturated (MUFA)/saturated fatty acid (SFA) and TRANS fatty acids in erythrocyte cell membranes was measured using omega index (OI) test (SanioTech) using GC-FID gas chromatography–flame ionization detector. To determined OI finger blood spots (~50 μL) was taken. OI measured in dried blood spots is a qualitative (%) expression of total fatty acids. Results: The participants had lower lever of: omega-3 (5.76 ± 1.4 vs. 7.9), EPA (1.1 ± 0.6 vs. 1.6), omega-6 (32.8 ± 3.1 vs. 35.3), MUFA (22.0 ± 2.1 vs. 28.9), SFA (38.6 ± 2.5 vs. 44.0) and Trans (0.57 ± 0.3) and higher AA (10.18 ± 1.4 vs. 8.1) compare to the mean value of references value. In addition, omega 6/omega 3 (AA/EPA) ratio was on 11:1. Conclusions: Despite the high awareness of the pro health influence of omega-3 and MUFA on muscle regeneration and immune regulation the participants did not consume appropriate amount and proportion of fatty acids with their diet.omega indexsocceromega 3

**Funding:** This research received no external funding.

## 56. Effects of Static and Dynamic Balance after Nine weeks of Whole-Body Vibration vs. Unstable-Surface Training in Postmenopausal Women

Del CerroNuria^1^*Rubio-AriasJacobo A.^2^AlcarazPedro E.^1^,^2^1UCAM Research Center for High Performance Sports, Catholic University of Murcia; UCAM, 30107 Murcia, Spain; palcaraz@ucam.edu2Faculty of Sport Sciences, Catholic University of Murcia, UCAM, 30107 Murcia, Spain; jararias@ucam.edu*Correspondence: ncerro@alu.ucam.eduDuring the postmenopausal stage in women, there is a bone demineralization that increases health expenditure due to fractures caused by falls. Thus, there is a growing need to create training protocols focused on one of the main factors that cause falls: loss of balance that leads to functional instability. The aim of this study was to analyze the effects of training in whole body vibration vs. unstable surface in the prevention of falls on the static and dynamic balance. We conducted a quasi-experimental design intra-and inter-subject with a control group of 39 sedentary postmenopausal women (58.8 ± 1.6 years). Participants were divided into three experimental groups and control group (G4). The experimental groups performed an incremental training, WBV (G1), unstable surface (G2) and floor (G3), for nine weeks three sessions/week. All participants maintained a static position of semi-squat and performed ankle plantar flexion. The only significant differences were observed between the groups G3 and G4. A decrease in the projection area in static balance was related to an improvement of the functional stability of the subject, and therefore, had more effect on postural control than any disturbance before the fall. Reducing the minimum contact time in dynamic balance is related to the speed of walk and a more reactive step and an improved ability to recover any disturbance of the postural stability. Therefore, the results obtained identify the motor pattern performed during training as a recommended exercise to reduce the projection area in static balance and the contact time in dynamic balance.balancewbvpostmenopausal women

**Funding:** This research received no external funding.

## 57. Postactivation Potentiation in Professional Football Athletes: The Effect of Physical Fitness Level

GuerraMauroJr.^1^CaldasLeonardo Carvalho^1^de SouzaHelder^1^TallisJason^2^DuncanMichael^1^Guimarães-FerreiraLucas^1^*1Federal University of Espirito Santo, Vitória, Brazil; guerrajr2@yahoo.com.br (M.G.J.); leocaldas03@gmail.com (L.C.C); helder_ldesouza@hotmail.com (H.S.); michael.duncan@coventry.ac.uk (M.C.)2Coventry University, UK; jason.tallis@coventry.ac.uk*Correspondence: lucas.ferreira@ufes.brTo investigate the relationship of the response to postactivation potentiation (PAP) with scores of physical fitness, 24 professional male soccer players undertook tests of agility, muscular power, aerobic capacity and body composition. Conditioning activities (CA) were performed consisting of plyometrics exercises and sprints with sled towing. In the first and second sessions, body composition, agility, power and aerobic capacity were assessed. At the third session, countermovement jumps (CMJ) were performed with one, three and five min after the execution of the CA. Significant differences were found for CMJ height one, three and five min after the conditioning activity compared to baseline values (3.58%, 5.10%, 5.48%, respectively). There was a significant positive correlation between the level of general physical fitness and PAP (CMJ height increase) 5 min post (r = 0.73). When the athletes were divided into groups with higher and lower physical fitness, the CA caused a significant increase in CMJ height in both groups, but a significant difference (*p* < 0.05) was observed at all times after PAP induction, with better performance in higher versus lower fitness level. The results suggest that a plyometrics exercises associated with sled towing sprints as a conditioning activity results in an increase in CMJ performance in athletes and that physical fitness directly influences the PAP occurrence, with higher fit players demonstrating an enhanced PAP response.postactivation potentiationphysical conditioningperformanceplyometrics

**Funding:** This research received no external funding.

## 58. Acute Effects of Cluster Training on Muscle Oxygen Saturation and Movement Velocity during Bench Press Exercise

Martínez-GuardadoIsmael*Mostazo-GuerraAlbertoGonzález-CustodioAdriánOlcinaGuillermoTimónRafaelGAEDAF Research Group, Faculty of Sport Science, University of Extremadura, Cáceres, Spain*Correspondence: imartinezg@unex.esThis study evaluates changes in muscle oxygen saturation and movement velocity comparing a traditional training with a cluster configuration during a barbell bench press exercise. A randomized crossover design was performed and 10 trained individuals (age: 24.1 ± 3.3; height: 173.3 ± 5.7; IMC: 22.9 ± 1.3) performed two resistance-training sessions under different protocols separated by at least 72 h: traditional (4 × 6 repetitions at 85% of 1RM with 120 s of recovery between sets) and cluster (4 × 2 + 2 + 2 repetitions at 85% of 1RM with 15 s of inter-set recovery and 90 s between sets). Physical performance was assessed by quantifying movement velocity using a linear position transducer that was fixed to the bar and muscle oxygenation level of the chest was evaluated using a portable NIRS device. No significant differences were found between training protocols. However, the accumulated muscular fatigue caused a higher loss of velocity (*p* < 0.05) in the 3rd (0.31 ± 0.09 m/s) and 4th (0.27 ± 0.09 m/s) set compared to the 1st (0.37 ± 0.07 m/s) and 2nd (0.33 ± 0.08 m/s) set, only in the traditional protocol. Although no significant differences were found between trainings, the proposed cluster protocol attenuated the velocity loss, which may have caused less fatigue during an upper body exercise.resistance exerciseintra-set restfatigue

**Funding:** This research was funded by the Government of Extremadura with funding from the European Regional Development Fund, Grant Number GR18003.

## 59. Effects of Six-Week Integrative Neuromuscular Warm-Up vs. FIFA11+ on Inter-Limb Asymmetries in Young Football Players

Marco-TomàsJ.^1^,^2^Beltrán-GarridoJ. V.^1^Romero-RodríguezD.^3^Madruga-PareraM.^1^,^2^,^3^*1University School of Health and Sport (EUSES), University of Rovira Virgili, Amposta, Spain2reQsport, Return to Play and Sports Training Center, Barcelona, Spain3University School of Health and Sport (EUSES), University of Girona, Girona, Spain*Correspondence: marcmparera@gmail.com; Tel.: +34-972-405-130The aim of this study was to compare the efficacy of two warm-up programs: integrative neuromuscular training (INT) and FIFA11+ on change of direction inter-limb asymmetries (COD_ASY_) and change of direction deficit inter-limb asymmetries (CODD_ASY_). Five young soccer teams (mean ± SD; age = 10.72 ± 0.72 years) were randomly allocated in either INT (n = 25) or FIFA11+ warm-up program (n = 19). Each group underwent two 20-min sessions per week for 6 weeks. A double 180° COD and 20-m sprint test were measured, and COD_ASY_ and CODD_ASY_ were computed prior to and at the end of the program. Neither INT nor FIFA11+ warm-up program affected COD_ASY_ (*p* = 1.000 and *p* = 0.145, respectively). On the other hand, FIFA11+ warm-up program increased the CODD asymmetries (*p* = 0.002), while INT warm up program did not affect this variable (*p* = 0.443).neuromuscular controlimbalancesmotor skills

**Funding:** This research received no external funding.

## 60. Effects of Different Ranges of Loads with the Same Mean Relative Intensity on Physical Performance

Sánchez-ValdepeñasJuan^1^*Riscart-LópezJavier^1^,^3^León-PradosJuan A.^1^,^2^Pareja-BlancoFernando^1^,^2^1Physical Performance and Athletic Research Center, Pablo de Olavide University, Seville, Spain2Department of Sports and Computing, Sport Faculty, Pablo de Olavide University, Seville, Spain3Department of Physical Education and Sports, University of Seville, Seville, Spain*Correspondence: juan_valdemate@hotmail.comThe effect of loading intensities during resistance training on physical performance has been analyzed without equalizing the efforts between ranges of load. The aim of this study was to analyze the effects on sprint, jump and squat strength gains by different ranges of loads (from 50 to 85% 1RM (R_50-85_, n = 12), from 55 to 75% 1RM (R_55-75_, n = 12) and from 60 to 70% 1RM (R_60-70_, n = 10)) with the same mean relative intensity (65% 1RM). Subjects followed an eight-week (twice per week) velocity-based training program using full squat. Pre- and post-training assessments included: 20-m sprint (T_10_, T_20_ and T_10-20_ (s)), countermovement jump height (CMJ (cm)) and a progressive loading tests in the SQ exercise (1RM (kg), average velocity attained against all absolute loads common to pre and post (AV, (m·s^−1^)). R_60-70_ showed significant differences in T_20_ and T_10-20_ (*p* ≤ 0.01 and *p* ≤ 0.001) and R_55-75_ in T_20_ (*p* ≤ 0.01) whereas R_50-85_ attained significantly greater improvements than R_60-70_ in AV (*p* ≤ 0.05). For that, ranges with low relative intensities could be better to improve sprint and ranges with heavier intensities could be more beneficial to improve squat performance in the entire range of loads (AV).velocity-based trainingrange of loadmean relative intensity

**Funding:** This research received no external funding.

## 61. Monitoring Wellness and External Load during a Major International Field Hockey Tournament

VarónPatricia^1^MorencosEsther^1^Romero-MoraledaBlanca^2^*1Faculty of Health Sciences, Universidad Francisco de Vitoria, Madrid, Spain; patricia.varon@ufv.es (P.V.); esther.morencos@ufv.es (E.M.)2Departamento de Educación Física, Deporte y Motricidad Humana, Universidad Autónoma de Madrid, Madrid, Spain*Correspondence: blanca.romero@uam.esField hockey consists of high-speed running, superimposed on low-speed periods of running where technical and tactical components are intertwined with changes of direction. World-class hockey tournaments often require the athletes to play an average of 5–8 games across a 10–12-day period, with limited recovery time. Time-motion analyses were conducted to assess whether the congested playing schedule and accumulated fatigue can result in reduced playing intensity across a tournament. The aim of the current observational study was to analyze the internal and external load of elite female field hockey players during a world class hockey tournament. Sixteen female field hockey outfield players’ external load (GPS) and internal load (psycho-metric questionnaire) data were recorded in all the competitive matches (7). The overall physical output showed no determined pattern throughout the competition. Results indicate that players were able to maintain intensity when playing seven matches in a period of fifteen days. Perception of all the wellness variables measured, excepting stress, increased as the end of the competition approached. Fatigue and muscle soreness data showed a constant decline, reflecting the load accumulation through the tournament. However, it was observed that a decrease in players’ daily well-being was not accompanied by changes in running performance in match-play.field hockeyexternal loadinternal load

**Funding:** This research received no external funding.

## 62. Effects of Different Velocity-Loss Magnitudes during the Set in the Bench-Press Exercise

Hidalgo-de MoraJavier^1^Cornejo-DazaPedro^1^Sánchez-ValdepeñasJuan^1^AlcazarJulián^2^Rodriguez-LopezCarlos^2^AlegreLuis M.^2^Sánchez-MorenoMiguel^3^Bachero-MenaBeatriz^3^Pareja-BlancoFernando^1^,^4^*1Physical and Athletic Performance Research Centre; Pablo de Olavide University, Seville, Spain2GENUD Toledo Research Group, Universidad de Castilla-La Mancha, Toledo, Spain3Department of Physical Educatio; University of Seville, Seville, Spain4Department of Sport; Pablo de Olavide University, Seville, Spain*Correspondence: fparbla@gmail.comEffects of different velocity loss (VL) on physical performance were analyzed on lower body, but there is not any study on upper body. Therefore, this study aimed to compare the effects of two resistance-training (RT) programs with different velocity loss (VL) thresholds: 0% (VL0) and 25% (VL25) on muscle structural and strength adaptations. Thirty young resistance-trained men were randomly assigned into two groups (VL0 and VL25) that differed in the VL allowed in each set. Subjects followed an RT program for eight weeks (twice per week) using the bench-press (BP) exercise, with similar relative intensity (70–85% 1RM), number of sets (3) and inter-set recovery period (four-min). Pre- and post-training tests included ultrasound images of pectoralis major for muscle cross-sectional area (CSA), maximum voluntary isometric contraction (MIF) in PB and progressive loading test in BP. Results showed a significantly higher increment (*p* = 0.008) in muscle CSA and greater gains in almost all strength variables analyzed for VL25 than VL0, although both groups improved its performance. In conclusion, VL near to half maximum possible repetitions in the set (25%) induces greater gains on muscle CSA and strength performance than a RT with minimum level of fatigue in BP (0%).velocity lossmuscle strengthbench press

**Funding:** This research received no external funding.

## 63. Determinants of Shooting Velocity in Elite Team Handball Players

KarcherClaude*PierratThibautSchoffitCarolineHureauThomas J.European Centre for Education, Research and Innovation in Exercise Physiology (CEERIPE, EA 3072), Sport sciences Faculty, University of Strasbourg, Strasbourg, France*Correspondence: claude.karcher@unistra.frBecause shooting velocity is a key factor to win games in handball, this study aimed to explore widely the determinants of shooting velocity. We measured upper- (n = 14), lower- (n = 20) body performances and anthropometric and mobility (n = 12) variables of elite young handball players (17.4 ± 2.9 years). Shooting velocities were collected in three different conditions (standing, run-up and jumping shots). To assess relations between velocities and performance determinants, we calculated the Pearson’s correlations coefficient between each variable and the three type of shots. Standing-shot velocity (90.5 ± 3.5 km·h^-1^) was largely related to body dimension (e.g., height, arm length). Large and very large relations exist between maximal bench pull (BPL, 76.7 ± 13.3 kg) and bench press (BPR, 77.6 ± 12.1 kg) strength. Run-up shots velocity (99.4 ± 6 km·h^-1^) was largely to very largely related to body dimension (e.g., weight) and to various to one leg dominant jump height, load maximal velocity in BPL and BPR. Jump shot velocities (92.1 ± 6.8 km·h^-1^) were largely related to body dimensions, sprinting speed and maximal velocity in BPL were largely to very largely linked to maximal strength in BPL and BPR. Our results showed that the upper-body strength plays a major role in shooting velocity. Notably, this study demonstrates for the first time that the bench-pull performance contributes to the same extent as bench press in shooting velocity.handball performancemuscle powerstrength and conditioningthrowing velocity

**Funding:** This research received no external funding.

## 64. Nutrition for Athletes according to Gut Microbiota: What We Need to Know

CasalsCristina^1^,^2^*Ponce-GonzálezJesús G^1^,^2^1MOVE-IT Research Group, Department of Physical Education, Faculty of Education Sciences, University of Cadiz, Cadiz, Spain2Biomedical Research and Innovation Institute of Cádiz (INiBICA) Research Unit, Puerta del Mar University Hospital, Cadiz, Spain*Correspondence: cristina.casals@uca.esIn this study, we review nutritional recommendations for athletes considering performance and health, specially focusing on combat sports in which rapid recovery is essential to perform several matches in one competition. These athletes compete according to body mass, promoting rapid weight loss through caloric restriction and intentioned dehydration, which compromises gut microbiota diversity influencing health, performance and behavior through the gut–brain axis that can modulate stress and anxiety, for example. Athletes may be able to improve reactive oxygen and nitrogen species (RONS) and inflammatory responses to exercise, reducing the recovery period, by improving gut microbiota diversity. Pre- and pro-biotics supplements may improve the metabolic function and immune system, while high-protein diets and protein supplements compromise the gut microbiota and impair fat-oxidation capacity, which results in higher body fat mass. However, the use of dietary protein supplements has shown contradictory results—especially in anaerobic sport modalities, which need to be more deeply studied. Finally, although some nutritional practices—as well as the use of antibiotics—can compromise gut microbiota, it was established that intense exercise can positively influence gut microbial diversity leading to improved health and performance through a complex mechanism with specific endocrine pathways expressing microbial gene products.microbiomedietcombat sportsperformancerecovery

**Funding:** This research received no external funding.

## 65. Acute Metabolic, Neuromuscular and Mechanical Response of Squat Exercise Protocols that Differ in Set Configuration

PiquerasFrancisco^1^*Pareja-BlancoFernando^1^GarcíaÓscar^2^1Faculty of Sports Science, Pablo de Olavide University, Seville, Spain; fparbla@gmail.com2Sport Performance, Physical Condition and Wellness Lab, University of Vigo, Pontevedra, Spain; oscargarcia@uvigo.es*Correspondence: piqueras.paco@gmail.comThe aim of this study was to compare the acute metabolic, neuromuscular and mechanical response of squat (SQ) exercise protocols that only differ in the set configuration (3 × 8 vs. 6 × 4). Sixteen moderately strength-trained male adults (mean ± SD; age: 23.4 ± 4.4 years; height: 17.9 ± 5.2 cm; body mass: 73.9 ± 9.1 kg) undertook a total of two sessions in the SQ exercise in a randomized order: session “A”—three sets of 8 repetitions with four-min inter-set rests (A3 × 8) and session “B”—6 sets of 4 repetitions with two-min inter-set rests (B6 × 4). Tensiomyography (TMG), Dm of VL and VM significantly decreased in VL and VM muscles for both protocols. Significantly higher reductions pre-post exercise in CMJ height was found for the 3 × 8 session compared to the 4 × 6 session (27.2% vs. 21.2%, *p* < 0.05). Blood lactate significantly increased for both conditions at post-exercise (*p* < 0.001), however, blood ammonia only increased for 3 × 8 condition, showing a significant protocol x time interaction (*p* < 0.05). From the results of this study, it seems evident that lower levels of velocity loss during the set allow athletes to perform the same total volume during a session but suffering lower levels of fatigue.tensiomyographysquat exercisemetabolic stress

**Funding:** This research received no external funding.

## 66. Lower Stimulus Lengths Increase Measurement Error between 10–18%: Methodological Implications for Tensiomyographic Measurements

PiquerasFrancisco^1^*Pareja-BlancoFernando^1^MartínSaúl^2^GarcíaÓscar^3^1Faculty of Sports Science, Pablo de Olavide University, Seville, Spain; fparbla@gmail.com2Department of Physical Education, University of Las Palmas, Gran Canaria, Spain; saulmrguez@gmail.com3Sport Performance, Physical Condition and Wellness Lab, University of Vigo, Pontevedra, Spain; oscargarcia@uvigo.es*Correspondence: piqueras.paco@gmail.comThe aim of this study was to determine the effect of changing the electrical stimulus length on muscle contractile properties assessment by tensiomyography (TMG). Thirty-six healthy young and moderately active volunteers (age 24.8 ± 5.8 years; height 178.2 ± 6 cm; weight 71.8 ± 7.3 kg; self-reported weekly moderate intensity activity 3.5 ± 1.2 h·week^−1^) were recruited for the study. A descriptive cross-sectional design was used in order to analyze the effect of different stimulus length on TMG parameters. Dm showed excellent relative (ICC 0.97–0.99 CI 95%) and absolute reliability (CV 2.9–4.1%) for all the stimulus lengths. The Dm increased by 10.4% (p = 0.001) between 0.2 and 0.5 ms, 7.0% (*p* = 0.001) between 0.5 and 1.0 ms and 18.2% between 0.2 and 1.0 ms (*p* = 0.001). The results obtained indicate that the longer stimulus lengths (up to 1 ms), the higher the Dm peak amplitude. This finding is methodologically relevant for stable TMG measurements since there are differences up to 18.2% between the minimum and maximus stimulus length of the TMG. On the other hand, this implies high measurement error if the stimulus length is not correctly chosen.tensiomyographystimulus length

**Funding:** This research received no external funding.

## 67. Dietary, Biomechanical, Biochemical and Thermographic Characterization for Performance and Injury Prevention of Amateur Master Athletes

Torres-PeraltaRafael^1^,^2^AlejosEva Gesteiro^1^,^2^Escobar-ToledoDavid^1^,^2^MartínezEnrique Díaz^1^,^3^Pantoja-ArevaloLisset^1^Calonge-PascualSergio^1^,^2^Sillero-QuintanaManuel^4^García-DíezEsther^1^UgarteMikel^4^BenítezJose^5^Guadalupe-GrauAmelia^1^,^2^González-GrossMarcela^2^,^6^*1ImFINE Research Group, Department of Health and Human Performance, Faculty of Physical Activity and Sport Sciences-INEF. Universidad Politécnica de Madrid, Madrid, Spain2Red Española de Investigación en Ejercicio Físico y Salud en Poblaciones Especiales (EXERNET), Spain3Laboratorio Clínico, Centro de Medicina del Deporte, Consejo Superior de Deportes, Madrid, Spain4Grupo de Análisis Biomecánico, Faculty of Physical Activity and Sport Sciences-INEF, Universidad Politécnica de Madrid, Spain5LFE Research Group, Department of Health and Human Performance, Faculty of Physical Activity and Sport Sciences-INEF. Universidad Politécnica de Madrid, Spain6Biomedical Research Centre of Pathophysiology of obesity and nutrition, CIBERobn, B12/03/30038, Carlos III Health Institute, Madrid, Spain*Correspondence: marcela.gonzalez.gross@upm.esThe number of master amateur athletes and of sport injuries is increasing due to physical activity promotion. The aim was to analyze usefulness of a specific protocol for injury prevention. Twenty-eight male mature amateur runners (48 ± 4.2 y., 174.1 ± 6.6 cm, 77.0 ± 8.3 kg, BMI 25.2 ± 2.0, fat mass 20.3 ± 5.9%, fat free mass 58.4 ± 4.8 kg, VO_2max_ 54.8 mL·kg·min^−1^) volunteered. Methods: body composition by DXA; gas exchange and maximal performance on a treadmill (IE); running biomechanics using VICON cameras; thermography images (IRT) of the legs; energy and nutrient content by two 24-h dietary recalls analyzed with DIAL©. Below recommendations for athletes were mean intake of energy (34 ± 7.2 kcal/kg), vitamin D (6.23 ± 9.13 µg), riboflavin (0.9 mg/1000 kcal), folic acid, magnesium and zinc. Caloric profile was 18.9 ± 3.7% protein, 40.8 ± 9.1% carbohydrates and 35.8 ± 8.4% lipids. Only total blood cholesterol was out of reference values. Running biomechanics showed no significant differences between the two measurements in the sagittal, frontal or lateral plane on both knee and ankle. Knee IRT decreased less than 1 °C in the frontal and slightly more on the posterior during IE. Diet and physical activity assessment—together with biomechanical running analysis and IRT of lower limbs—could complete the information provided leading to a better manage of injury prevention.athletic injuriesmicronutrientsthermography

**Funding:** This research was funded by Natural Origins, through an agreement with Universidad Politécnica de Madrid, Grant Number P1811600374. The funding sponsor had no role in the design of the study, the collection, analysis nor interpretation of the data, the writing of the manuscript or in the decision to publish the results.

**Conflicts of Interest:** The authors declare no conflict of interest.

